# Bacterial R-bodies with common morphologies and unrolling dynamics are phylogenetically scattered, indicating extensive lateral gene transfer and wide application potential

**DOI:** 10.1128/aem.00864-26

**Published:** 2026-09-11

**Authors:** Lennart Dörr, Robin Ghosh, Michael Schweikert

**Affiliations:** 1 Department of Biobased Materials, Institute of Biomaterials and Biomolecular Systems, University of Stuttgart 9149, Stuttgart, Germany; 2 Department of Bioenergetics, Institute of Biomaterials and Biomolecular Systems, University of Stuttgart, Stuttgart, Germany; 3 SRF Advanced Materials Innovation and Characterization (AMICA), University of Stuttgart 9149, Stuttgart, Germany; Michigan State University, East Lansing, Michigan, USA

**Keywords:** R-bodies, inclusion bodies, gram-negative bacteria, electron microscopy, bacterial morphology, phylogenetics

## Abstract

Refractile bodies (R-bodies) of gram-negative bacteria are large proteinaceous assemblies, rolled up in the form of an Archimedean spiral. They exhibit rapid rod-like reversible extension in the micrometer range when cued by chemical environmental triggers and have potential for synthetic biology and biochip applications. Initially described for the *Paramecium* endosymbionts *Caedibacter taeniospiralis* and *Caedimonas varicaedens*, R-bodies have since been discovered in many classes of Pseudomonadota, both in endosymbionts and in non-endosymbionts. However, despite the fact that the genetics and morphologies, as well as the unrolling kinetics of R-bodies from different species, show considerable diversity, no recent study has integrated these aspects into a single framework. The latter would be advantageous for the creation of an R-body biotechnology toolbox, where different properties determine the application area. Here, we have examined the R-bodies from six different Pseudomonadota, comprising both phylogenetically diverse endosymbionts and non-endosymbionts. Comparison of the morphologies of the rolled-up and unrolled forms, obtained using electron microscopy and high-quality images, to their corresponding genetic data indicates that extensive lateral gene transfer has occurred, which confounds a common framework based on these data. However, we have also studied the R-body extension and retraction kinetics using high frame-rate light microscopic video recordings, where we show for the first time that R-bodies can be classified into two classes, showing “fast burst” or “slow” acid-induced extension kinetics, respectively. We propose that this criterion may, in fact, be the most useful for the choice of an R-body tool for biotechnological purposes.

**IMPORTANCE:**

R-bodies are unique proteinaceous macromolecular structures capable of massive reversible extension in response to external environmental triggers without the input of chemical energy. They comprise only a few small polypeptides, which makes them potentially highly amenable to tuning via genetic engineering, as well as being exceptionally stable. These properties would be highly desirable in biotechnology and synthetic biology, as well as in biochip applications, where a controlled mechanical extensor might play an integral part in a nanoscale molecular machine. So far, only R-bodies from a single species, *Caedibacter taeniospiralis*, have been characterized extensively. However, in recent years, genomic information has revealed that a panoply of R-bodies are widely distributed among gram-negative phyla, although studies have generally not included morphological data. This study brings these two areas together to provide a holistic overview of the field and also reveals new insights into key dynamic aspects of R-body extension.

## INTRODUCTION

Refractile bodies (R-bodies), found in a wide variety of gram-negative bacteria, are a fascinating type of intracellular inclusion body with nanomachine extensor properties (1, 2), which show promise as a molecular tool for biotechnology, synthetic biology, and biochip applications (3, 4).

R-bodies were originally identified in a bacterial endosymbiont that confers a killer trait to paramecia and were observed as spirally rolled ribbons within the bacterial cytoplasm (5, 6). Usually, only a single R-body, which is predominantly proteinaceous in nature (7), is present and occupies a large volume of the bacterial cytoplasm (6). Typically, isolated R-bodies exhibit an intriguing property that, when subject to chemical environmental triggers (e.g., a decrease in pH), show unwinding and extension to generate a helical filamentous form that exceeds the length of the folded R-body by more than 20-fold. The unfolding transition is completely reversible and remains operational even after the R-bodies are subjected to strong, potentially denaturing, chemical treatments (1, 6, 8–10).

In nature, the extension property of R-bodies in endosymbiotic (ES) bacteria is partially responsible for the “killer” effect. Paramecia with R-body-producing endosymbionts occasionally release their symbionts into the environment through exocytosis (11, 12). When an R-body-harboring bacterium is digested in the food vacuole after phagocytosis, the acidic pH causes the R-body to unroll, thereby killing the sensitive *Paramecium* through destruction of cellular integrity (13–16). The killing mechanism is still unclear, but it has been suggested that an unknown toxin may be delivered through the R-body, though experimental evidence for this suggestion is still lacking (6, 17).

Much less is known about the role of R-bodies in bacteria that are not endosymbionts of *Paramecium* spp., although some “killer” effects have still been documented. For instance, it has been shown that the R-body-containing symbiont *Azorhizobium caulinodans* present in root cells of the legume *Sesbania rostrata* may exhibit a “killer” effect after acidification of the peribacterial space during nodule morphogenesis (18, 19). It has also been shown that R-bodies may act as virulence factors or play a role in host colonization (20). In fact, an early and pioneering phylogenetic analysis (21) revealed that R-bodies are probably widely distributed in bacteria (discussed in more detail below).

It was recognized early (see Pond et al. [1] for a review) that R-bodies are not uniform in either their morphology or unfolding behavior. On the basis of five types of R-bodies, Pond et al. (1) suggested that R-bodies could be classified into distinct types (51, 7, Cc, Ps, and Pa) defined in terms of length and width of the extended ribbon, as well as ribbon shape, end geometry, and the topological unfolding mechanism. These included strains from *Paramecium* endosymbionts *Caedibacter taeniospiralis* (Type 51) and *Caedimonas varicaedens* (Types 7 and Cc, see Schrallhammer et al. [22]), as well as the non-endosymbionts *Acidovorax avenae* (Type Pa) and *Hydrogenophaga taeniospiralis* (Type Pt).

According to Pond et al. (1), Type 51 and Type 7 R-bodies have an extended length of about 20 µm and a ribbon roll-up width of about 0.4 µm. However, whereas both ends of the Type 51 R-bodies appear tapered, only the outer terminus of the Type 7 R-bodies is tapered, with the inner terminus appearing “blunt.” Type 51 R-bodies appeared to unroll from the inside (here, we designate this unrolling mechanism, also called “telescopic,” as “inside-unrolling [i-u]”), whereas Type 7 R-bodies were suggested to unroll from the outside (“outside-unrolling [o-u]”).

Pond et al. (1) also noted that the response of the R-body types to external environmental triggers also differed. Whereas Type 51 R-bodies unfolded in response to lowering the pH below 6.5, Type 7 R-bodies appeared to show no response to a pH decrease. Another fundamental difference in the two types of R-bodies is that Type 7 R-bodies were reported to be surrounded by a membranous sheath, whereas this is not observed for Type 51 R-bodies (7). Type Cc R-bodies seemed to be a variant of Type 7 R-bodies, with a broader roll-up width (~0.8 mm) and blunt and tapered outer and inner ends, respectively.

Pt and Pa R-bodies showed significant differences from the above types and from each other. Whereas the width of the extended ribbon for Pt R-bodies was far narrower (~0.25 µm) and also shorter (<10 µm) than that mentioned above, the corresponding width of Pa R-bodies was similar in roll-up width to Type Cc R-bodies and was significantly longer (~30 µm). The ends of Pt R-bodies are analogous in shape to Type 7 R-bodies (outer end blunt, inner end tapered), whereas both ends of the Pa R-bodies are long and tapered. Pond et al. (1) also documented that Pt R-bodies unroll via an i-u mechanism, whereas Pa R-bodies appeared to exhibit o-u unrolling.

One might expect that the heterogeneity in morphologies and unrolling mechanisms would be reflected in the sequence and genetic organization of R-body polypeptide-encoding *reb* genes in the respective organisms. However, establishing this relation has proved to be elusive, largely due to the unexpected variations in *reb* gene organization and composition (21), as well as the difficulty in doing genetic manipulation in many R-body-containing organisms. At present, the *reb* genes that encode the Type 51 R-bodies in *Cb. taeniospiralis* are the most well studied. It was discovered early (23–26) that *Cb. taeniospiralis* maintains a natural plasmid, which encodes a 1.8 kb operon of four genes (*rebABCD*) (26, 27). Expression of the *rebABCD* operon in *Escherichia coli* allows the production of R-body-like structures, which appear morphologically identical to those observed in *Cb. taeniospiralis*, but do not exhibit a “killer” trait (4, 9, 17, 25–27). In general, the *reb* genes encode small polypeptides with molecular masses varying from 7 to 15 kDa. It has been suggested that RebA and RebB are the major structural components of the *Cb. taeniospiralis* R-body, whereas RebC is a “modifying” protein (27). The role of *rebD* is presently unknown. However, we note that the functional assignments of the *Cb. taeniospiralis reb* genes are still tentative and, in view of the weak homologies between Reb proteins in different organisms, may not be general (see Discussion).

In 2013, Raymann et al. (21) presented the first extensive phylogenetic analysis for the distribution of Reb protein homologs in Pseudomonadota. Using the *Cb. taeniospiralis* RebABCD proteins as starting search “seeds,” they were able to identify 203 Reb protein homologs. They could show that not only are Reb protein homologs present in most classes of Pseudomonadota, as well as in one species (*Kordia algicida* OT-1) belonging to the Bacteroidota phylum, but they also showed for the first time that some bacteria have multiple copies of *reb* gene clusters within their genomes. The presence of multiple *reb* gene clusters in a single species has also been confirmed recently for some species belonging to the order Holosporales (28). In many of these species, R-bodies have not been observed experimentally (e.g., reference 29), although this may be due to the fact that particular induction conditions may be necessary to induce *reb* gene expression (20, 28).

The more recent phylogenetic studies above, as well as our own (see below), have shown that different bacterial classes seem to possess a characteristic order and clustering of *reb* genes, which has led us to ask whether this might be reflected in the overall R-body morphologies observed. A correlation between genetic arrangement and morphology would imply a temporal dimension to the *reb* gene expression. In this study, we examine the connection between phylogenetic classification and overall morphology or dynamic behavior, in particular, different modes of shape transition and induction of unrolling triggered by environmental changes (1, 6, 10, 30–34).

To aid this analysis, we have included a preliminary phylogenetic outline, where we focus on seven types of R-bodies that have been demonstrated experimentally. These include both obligate endosymbiotic and non-endosymbiotic (NES), α-, β-, and γ-proteobacteria (PB): *Cb. taeniospiralis* (ES, γ-PB), *Cm. varicaedens* (ES, α-PB), *Roseibium alexandrii* (NES, α-PB), *Ac. avenae* (NES, β-PB), *Hy. taeniospiralis* (NES, β-PB), and *Marinomonas mediterranea* (NES, γ-PB). Our analysis also includes *Pseudomonas chlororaphis* (NES, γ-PB) that was previously not known to produce R-bodies. *Ps. chlororaphis* has been suggested to be potentially useful as a biopesticide (35).

In a complementary corollary to both the transmission electron microscope (TEM) images and the phylogenetics, we have also defined the unrolling dynamics of the isolated R-bodies from the respective species (with the exception of *Cb. taeniospiralis*, which has been extensively studied previously) and examined possible relationships (if any) with their phylogenetic classification.

## RESULTS

### Comparison of R-body induction and isolation procedures for the various strains

We observed that R-body production varies between species and can, in some cases, be increased through different stressors. The *Paramecium* endosymbiont *Cm. varicaedens* exhibited only low levels of R-bodies when cultured under nutrient-rich conditions, whereas R-body production was considerably enhanced when grown with nutrient limitation. In general, the presence of R-bodies in all NES cells tested here was drastically lower than that observed for *Cm. varicaedens* but could also be increased under conditions of nutrient limitation. Another interesting observation is that the growth temperature affected R-body formation, with lower temperatures being correlated with an increase in R-body production. In addition, R-body production was enhanced in *Ro. alexandrii* as a response to a short UV-A light exposure of plate-grown cells; no UV effect was observed for other NES strains. Interestingly, TEM images of isolated R-bodies from *Ps. chlororaphis*, detected here for the first time, also revealed a large number of tubular, phage-like structures (Fig. S2A) that bear resemblance to “rhapidosomes” or contractile injection systems (36–39).

Finally, the use of the detergent sodium dodecyl sulfate (SDS) during R-body isolation proved highly effective in both increasing R-body yield and purity when compared to Triton X-100. However, since SDS can cause unrolling of R-bodies, we tried avoiding its use in our isolation protocols. However, whereas we could successfully substitute octyl-polyoxyethylene (OPOE) or Triton X-100 for SDS during R-body isolation from *Cm. varicaedens*, *Ro. alexandrii*, *Ac. avenae,* and *Ps. chlororaphis*, we were unable to isolate useful amounts of R-bodies from *Hy. taeniospiralis* and *Ma. mediterranea* without SDS.

### Comparative morphology and unrolling mode analysis of R-bodies from different species

The early morphological classification of R-bodies by Pond et al. (1) relied on essentially four bacterial species: *Cb. taeniospiralis*, *Cm. varicaedens* (the current synonym of *Cb. caryophilus*, based on 16S rRNA gene sequences; see Schrallhammer et al. [22]), *Hy. taeniospiralis*, and *Ac. avenae*. Here, we have re-examined the R-body morphology of the former species (with the exception of *Cb. taeniospiralis*, which has been well-documented elsewhere [1, 6, 12, 14, 30, 34, 40]), as well as that from three further species, using a modern high-voltage transmission electron microscope for rolled-up isolated R-bodies and high-pressure freezing cryo-fixation to show cellular R-bodies on ultra-thin sections.

**Fig 1:**
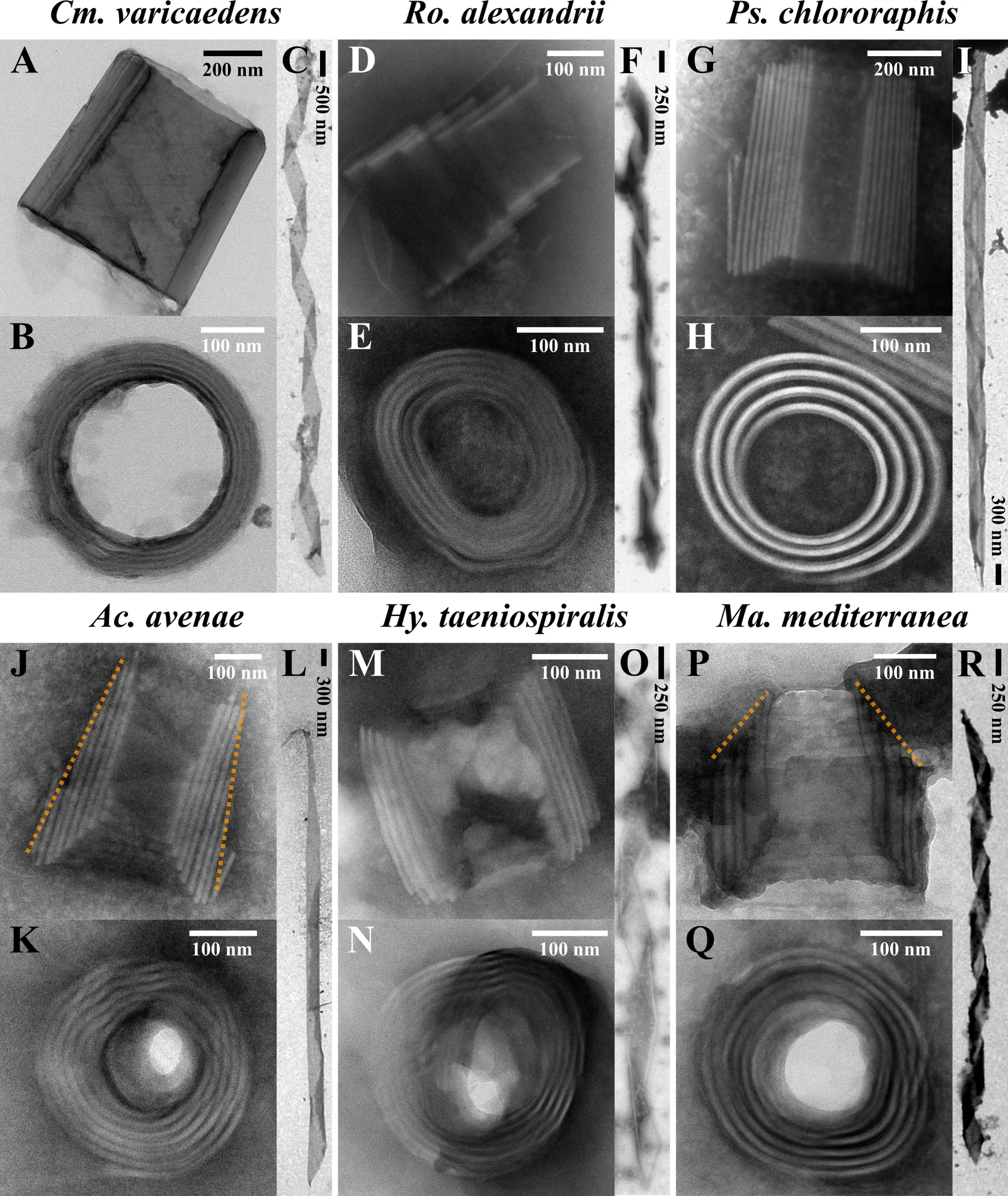
Negative contrast transmission electron microscopy images of isolated R-bodies of various bacterial species. (**A–C**) The three panels for each species show a side view of rolled-up R-bodies (**A, G, J, and M**), a side view of partially unrolled R-body from the side (**D and P**), rolled-up R-bodies from the top in panels **B**, **E**, **H**, **K**, **N**, and **Q**, as well as R-bodies unrolled through HCl in panels **C**, **F,** and **I** or unrolled after isolation in panels **L**, **O,** and **R**. The “Pan flute” geometry of the rolled-up forms is highlighted with dashed orange lines.

We confirm that all of the R-bodies studied exhibit an “Archimedean spiral” morphology (Fig. 1A, B, D, E, G, H, J, K, M, N, P, and Q), although the dimensions observed can vary considerably between species. Our TEM images confirm that the R-bodies from *Cm. varicaedens*, *Hy. taeniospiralis*, and *Ac. avenae* show morphologies corresponding to Type 7 (large, outer end/inner end: blunt [b]/tapered [t] [Fig. 1C]), Type Pt (short-narrow, outer end/inner end: b/t [Fig. 1O]), and Type Pa (short, narrow, outer end/inner end: t/t [Fig. 1L]), respectively, as previously reported (1). For the additional species, *Ro. alexandrii*, *Ps. chlororaphis,* and *Ma. mediterranea*, studied here for the first time, the R-bodies seem to be similar to Type 51 (ra, pc, and mm) ribbon shapes (Fig. 1F, I, and R). However, the extended lengths of all R-bodies reported here are lower than those reported previously (1). We cannot explain this discrepancy with the previous literature, although a strain difference cannot be excluded.

Closer perusal of the TEM images reveals that, whereas the lamellar side-view projections of the rolled-up forms of most R-bodies are approximately of equal length (Fig. 1A, D, and J), those of *Ac. avenae* and *Ma. mediterranea* have a “Pan flute” appearance (Fig. 1M and P), where the outer lamellar layers appear incrementally shortened with respect to the inner ones.

For all R-body types, except those from *Ac. avenae,* we observed i-u-type telescopic unrolling consistent with the literature (1).

Most unrolled R-bodies exhibited a characteristic “zigzag” appearance in TEM images, suggesting that the rings of the rolled-up Archimedean spiral separate during extension. Some of the extended R-bodies of *Ps. chlororaphis* showed continuity between the zigzag lamellar repeats (Fig. 1I). This is likely caused by some of the *Ps. chlororaphis* R-bodies having an unusually large barrel height (e.g., Fig. S2B). Interestingly, the repeat length per turn still shows a direct relation (described by a second-order polynomial, see below) to the total extended length.

**Fig 2:**
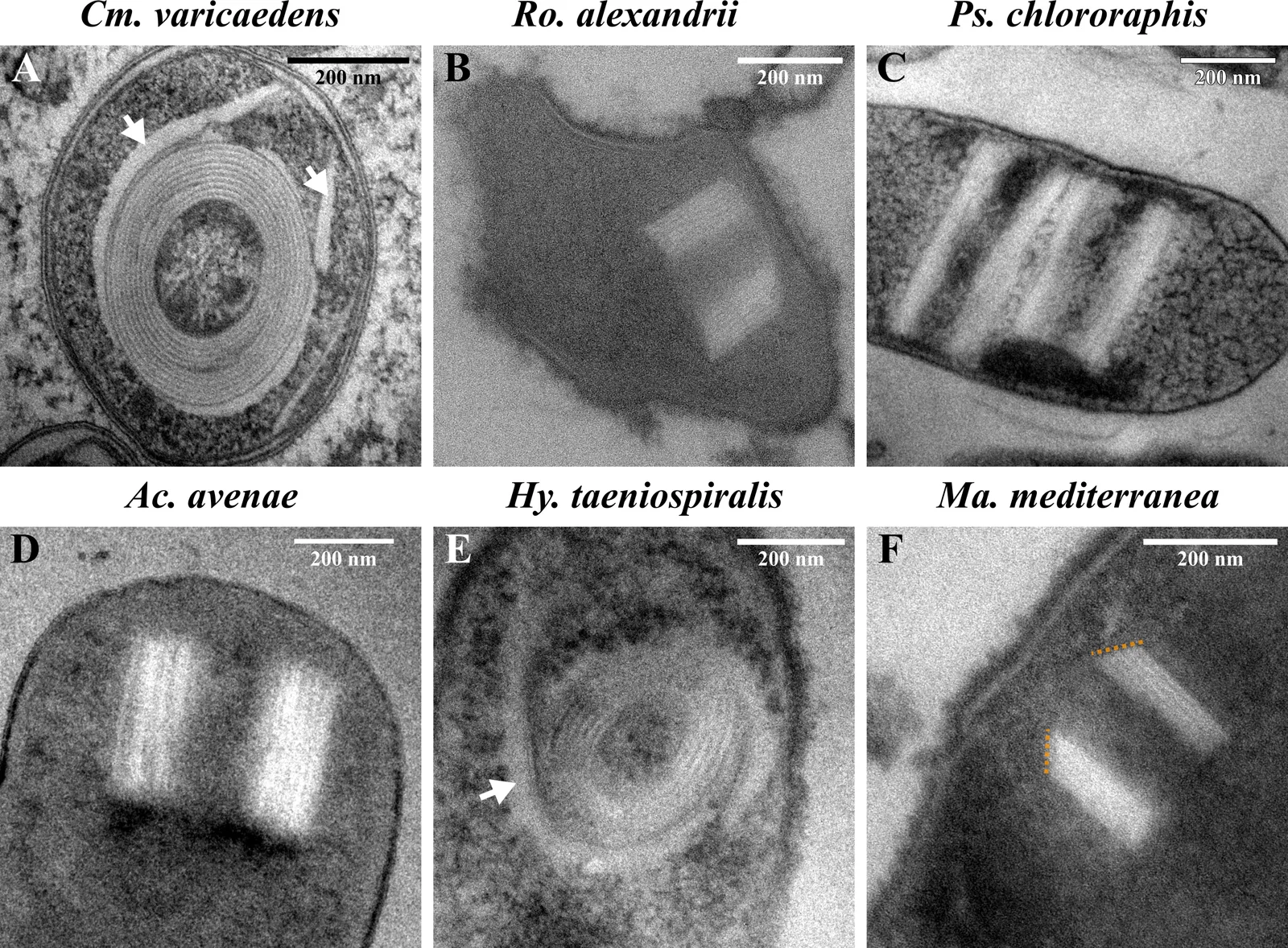
Transmission electron microscopy images of ultra-thin sections showing R-bodies within various bacterial cells after high-pressure freezing. (**A**) *Cm. varicaedens* in the macronucleus of *Paramecium caudatum*, (**B**) *Ro. alexandrii*, (**C**) *Ps. chlororaphis*, (**D**) *Ac. avenae*, (**E**) *Hy. taeniospiralis*, and (**F**) *Ma. mediterranea*. For *Cm. varicaedens* and *Hy. taeniospiralis*, the bright membrane sheath has been highlighted with white arrows (see also Fig. S2). The “Pan flute” geometry of the rolled-up forms is highlighted with dashed orange lines.

It is instructive to compare the morphology of isolated R-bodies to those observed in intact cells, as visualized by TEM of ultra-thin sections (Fig. 2). In particular, the TEM images from ultra-thin sections of *Cm. varicaedens* and *Hy. taeniospiralis* exhibit bright structures around the R-body (Fig. 2A andE , respectively, and Fig. S2D through F, respectively), which are consistent with the interpretation of a membranous sheath previously observed for *Cm. varicaedens* (41). Some of our images also indicated that the sheath or outer lamella is connected to the bacterial membrane (Fig. S2D through F). Since the cells were high-pressure frozen, it is unlikely that these sheath-like structures are artifacts caused by local dehydration or chemical reactions. However, we never observed such prominent sheath-like structures for the R-bodies of the other organisms. Finally, for *Ps. chlororaphis*, we also observed that two R-bodies were “paired” within a single cell (see Fig. 2C), as has also been previously documented for other species (12, 42). The morphology of *Ps. chlororaphis* R-bodies closely resembles that previously described for those of *Ps. aeruginosa* PA14 (20, 43).

### Analysis of the extension/retraction dynamics of R-bodies from different species

**Fig 3:**
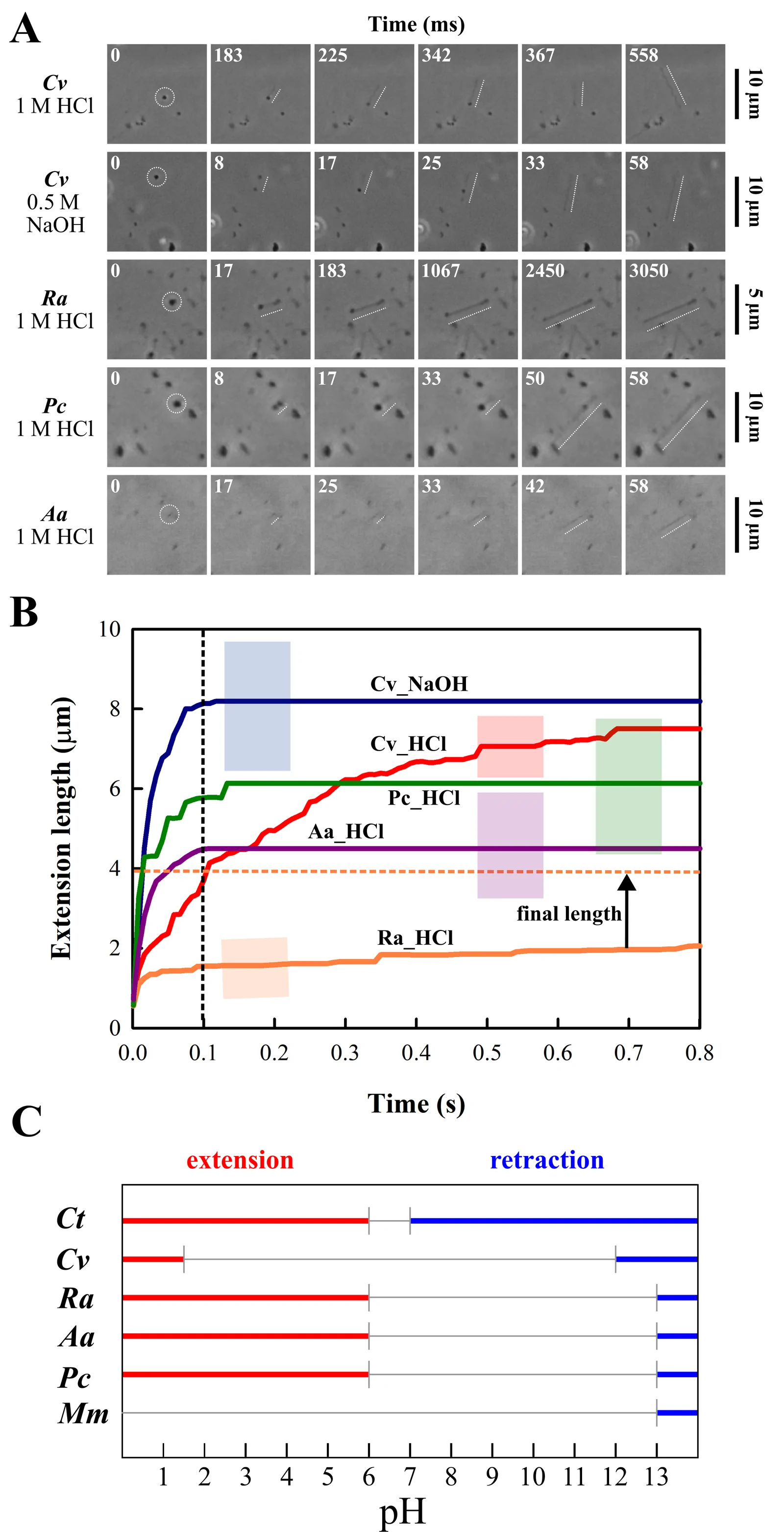
Phase-contrast light microscopy imaging and analysis of isolated R-bodies: (**A**) cropped single frames of R-body unrolling of various bacteria due to either NaOH- or HCl-mediated unrolling initiation. Concentrations: HCl, 1 M; NaOH, 0.5 M. Brightness and contrast were adjusted to facilitate visual clarity. The white dotted line placed next to the extending R-body has the same length as the observed structure. *Cv*, *Cm. varicaedens; Ra*, *Ro. alexandrii*; *Pc*, *Ps. chlororaphis*; and *Aa*, *Ac. avenae*. (**B**) Summary of the pH-induced kinetics of R-body unrolling. Solid lines, averaged data; shaded boxes, sampling of the standard deviation range of each data set. The black dashed line indicates the 100 ms time point for the final extension length of “fast burst” R-bodies. The final extension length of R-bodies from *Ro. alexandrii* is also shown (orange dashed line). (**C**) The pH ranges at which R-bodies of various bacteria unroll (extend) and retract. The pH range for *Cb. taeniospiralis* was obtained from reference 6.

The extension/retraction dynamics of R-bodies from *Cm. varicaedens*, *Ro. alexandrii*, *Ps. chlororaphis*, and *Ac. avenae* induced by either an acid or alkali transition were examined by high-speed imaging using light microscopy. In each case, the lengths of unrolling R-bodies were determined frame by frame (see individual panels in Fig. 3A) until the final unrolled extension length was reached. For all cases, at least nine longitudinal extension events were averaged. We note that since R-bodies from *Hy. taeniospiralis* and *Ma. mediterranea* were only obtainable in low yield and purity, they were not suitable for high-speed light microscopic imaging. Video recordings of the unrolling process can be viewed in the supplemental material.

Consistent with previous observations with the isolated R-bodies from *Cb. taeniospiralis* (4, 6, 17, 30), R-bodies from *Cm. varicaedens*, *Ro. alexandrii*, *Ps. chlororaphis*, and *Ac. avenae* all exhibited low pH-induced unrolling events (Fig. 3A), which could be reversed by subsequent elevation of the pH by the addition of alkali. To induce the retraction of the R-body, the pH needed to be increased far beyond the original pH required for unrolling in all cases. For the R-bodies from *Ro. alexandrii*, *Ps. chlororaphis*, and *Ac. avenae*, the low pH unrolling point was determined to be pH 6.0, whereas pH 13.0 was required to initiate R-body retraction. Interestingly, the unrolling of R-bodies from *Cm. varicaedens* exhibited a significantly lower pH transition point (between pH 1.0 and 2.0 depending on individual R-body), although the alkaline retraction point (pH ≥12.0) was similar to those described above. For R-bodies from *Hy. taeniospiralis* and *Ma. mediterranea*, TEM images of negatively stained samples after treatment with 0.5 M HCl showed only unrolled forms, consistent with acid-induced extension. The pH range for those types of R-bodies could not be determined due to the low yield and purity of the isolates. However, whereas most acid-treated R-bodies could be reversibly retracted by the addition of alkali (0.1 M NaOH), we observed that the corresponding retraction process observed for the R-bodies from *Ma. mediterranea* at pH ≥13.0 was irreversible.

In contrast to the above observation of acid-alkali-induced unrolling-retraction events, which have also been reported previously (4, 6, 30), we also observed a new environmental trigger for this phenomenon. Isolated R-bodies from *Cm. varicaedens* could also be induced to unroll by NaOH concentrations as low as 5 mM. However, in contrast to acid-induced reversible unrolling, the NaOH-induced unrolling event is only transient, and the extended R-bodies retracted again within a short time period even though no further environmental change was initiated. The NaOH-induced extension-retraction transition was not inducible repeatedly by the further addition of NaOH. This effect has not been documented previously. We note, however, that in many cases, R-bodies of *Cm. varicaedens* did not fully return to their rolled-up state after alkali-induced unrolling.

In general, we observed that isolated R-bodies of *Cm. varicaedens* were less likely to show unrolling events after long-term storage at 4°C but retained their unrolling potential when stored at −80°C.

Figure 3B summarizes the pH-induced extension kinetics of R-body unrolling observed by high-speed light microscopy imaging. Particularly striking is the observation that the final extension length of reversibly unrolled R-bodies from *Ac. avenae*, as well as *Ps. chlororaphis,* is shown as a rapid “burst,” which is completed at about 100 ms, whereas the low pH unrolling of R-bodies from *Cm. varicaedens* is about four times slower. In contrast, the NaOH-induced unrolling of R-bodies from *Cm. varicaedens* is also complete after about 100 ms. The low pH-induced unrolling kinetics of R-bodies from *Ro. alexandrii* were also slow, where the unrolling was only complete at about 3 s. Taken together, these data would indicate that the unrolling kinetics are not associated with a particular R-body morphology but must be determined on a case-by-case basis. We could also confirm previous observations of SDS-induced unrolling of *Cm. varicaedens* (6) and discovered that this is also true for R-bodies from *Ac. avenae*. However, heterogeneity of the data for SDS-induced unrolling of *Cm. varicaedens* R-bodies prevented precise quantification. In addition, R-body retraction kinetics were not determined due to technical limitations of our microscope setup.

Finally, analysis of the pH ranges for inducing R-body extension and retraction, respectively, for various species, reveals two interesting differences (Fig. 3C). First, the pH range for R-body extension from all species, with the exception of that from *Cm. varicaedens*, is broad (Fig. 3C) and strikingly similar to that previously reported for *Cb. taeniospiralis* (6, 30). Second, and in contrast to the above, for most species (with the exception of *Cb. taeniospiralis*), the pH range for the induction of R-body retraction is very small (Fig. 3C). The larger pH range observed for *Cb. taeniospiralis* has been reported previously (6, 30).

### Preliminary phylogenetic outline of the *reb* operons in this study

**Fig 4:**
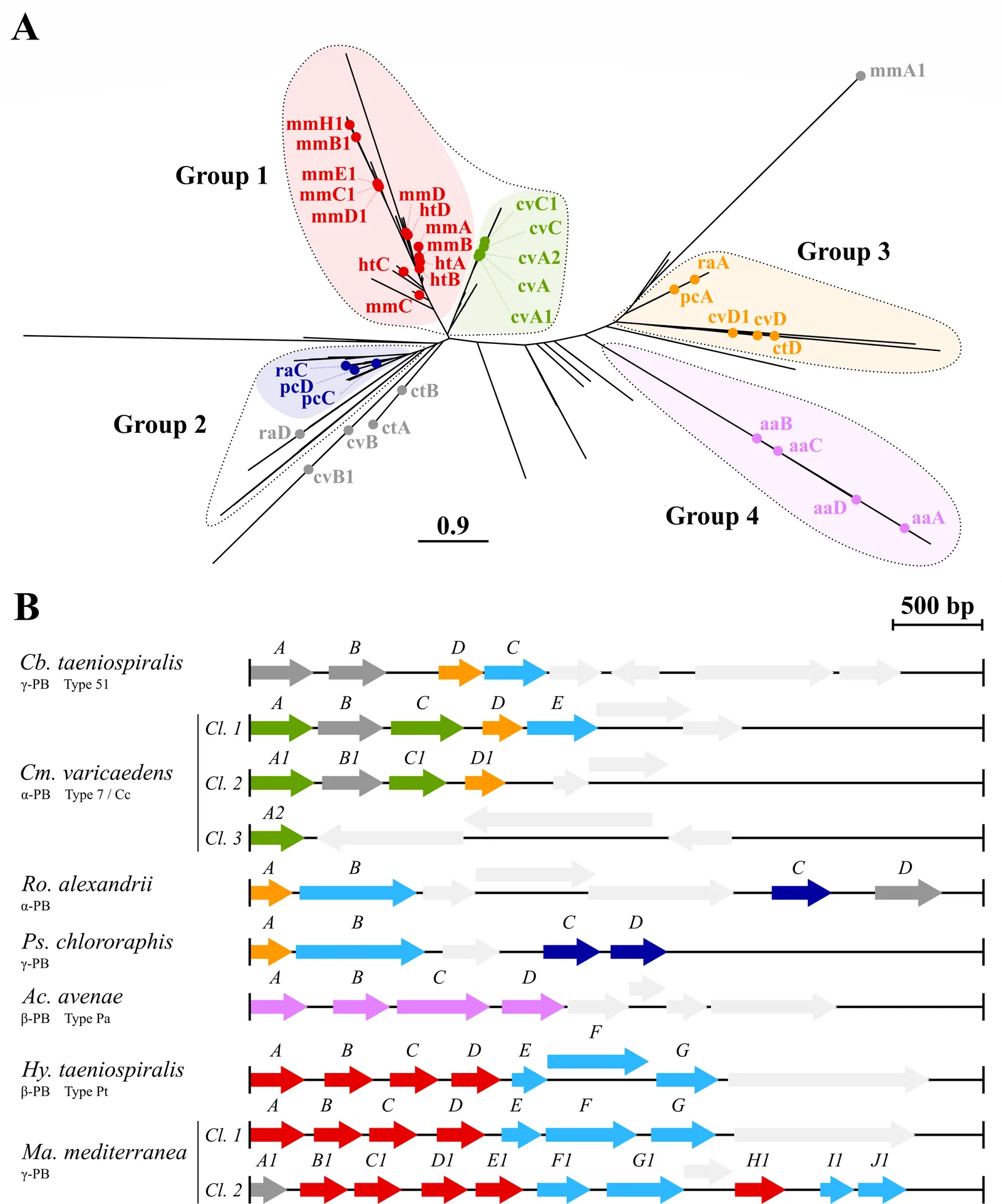
(**A**) Unrooted phylogenetic maximum-likelihood tree based on sequences of 56 different Reb proteins showing homology to ctRebB. Scale bar: the estimated proportional sequence divergence. ct, *Cb. taeniospiralis*; *cv*, *Cm. varicaedens*; *ra*, *Ro. alexandrii*; *pc*, *Ps. chlororaphis*; *aa*, *Ac. avenae*; *ht, Hy. taeniospiralis*; and *mm*, *Ma. mediterranea*. For labeling of proteins, “Reb” was omitted for readability (e.g., cvA is cvRebA). (**B**) *reb* operon structure of the species compared in this study. Proteobacterial class and R-body type (1) are indicated. Color code for panel A matches the corresponding genes in panel B. Dark gray, Reb sequences with generally unstable placement on the tree; *reb*-related, light blue; and unrelated, light gray.

Figure 4A shows the phylogenetic consensus tree, derived from an initial data set of RebB homologs found in 56 bacterial strains (see Materials and Methods for details). The final tree analysis focuses on the Reb proteins studied here, for which we also have experimental morphological data. Our phylogenetic outline, which has focused on defining regions where Reb proteins group together, indicates that the Reb proteins in this study fall into four distinct groups (Groups 1–4), where Groups 1 and 2 contain subgroups. These groupings are further supported by the highly similar respective gene syntenies of the *reb* clusters of *Ro. alexandrii* and *Ps. chlororaphis*, as well as of *Hy. taeniospiralis* and *Ma. mediterranea*. Interestingly, the RebA (maA1) from *Ma. mediterranea* seems to be an outlier, showing only weak homologies to the other Reb proteins studied here. In general, however, it is striking that whereas Reb proteins from some strains (*Ac. avenae* and *Ma. mediterranea*) appear to cluster mainly within the same group (or subgroup, see Group 1 in Fig. 4) (cf. Groups 4 and 1, respectively), the Reb proteins of others are more “heterogeneous.” In particular, RebA from *Ro. alexandrii* and *Ps. chlororaphis* (raRebA and pcRebA) cluster more closely to RebD from *Cm. varicaedens* (cvRebD), whereas RebC and RebD from the aforementioned strains (raRebC, raRebD, pcRebC, and pcRebD) cluster more closely with RebA and RebB from *Cb. taeniospiralis* (ctRebA and ctRebB).

Examination of the genome organization of the Reb proteins studied here is both complementary to the phylogenetic analysis and also yields more intriguing details (Fig. 4B). First, most Reb proteins are arranged in operon-like structures, which can differ significantly in their details. For instance, whereas the *reb* regions of *Cb. taeniospiralis* and *Cm. varicaedens* are visually similar, they appear significantly different from those of the other strains.

In their early analysis, Raymann et al. (21) assumed that the four *Cb. taeniospiralis* RebABDC genes composed a paradigmatic “*reb* cluster,” which allowed them to define surrounding genes with little or no homology to ctRebB as “Group 1 and 2 synteny” genes (those with low homology to Reb genes are shown in blue in Fig. 4B). In fact, closer analysis of the amino acid sequences in Fig. 4A shows that this definition is misleading. First, the ctRebABDC gene products are not optimal as a paradigm, as they show only partial similarities to their closest “relatives” in our alignment. For instance, whereas *Cb. taeniospiralis* RebA (here called ctRebA) and RebB (here ctRebB) show a high level of identity (46.6%) to each other and significantly less to RebC (here ctRebC) (28.3%), RebA (cvRebA) and RebC (cvRebC) from *Cm. varicaedens* exhibit only weak identity (32.8%), but cvRebA shows a high level of identity (61.9%) to cvRebC. Thus, for this example, cvRebC may possibly be functionally equivalent to ctRebB. A similar argument can be made for genes in other clusters. For instance, although the raRebB and pcRebB genes are placed into the “*reb*-related” synteny class, they are not only closely homologous to each other (67.9% identity) but are also strikingly homologous to ctRebC (33% and 35% identity, respectively). Our overview of different *reb* gene clusters (Fig. 4B) further shows that the genes of *Cb. taeniospiralis* are also not optimal as a paradigm with regard to their chromosomal *reb* gene organization and that a *rebD*-like gene must not necessarily be present on the *reb* clusters of other species.

It is also striking that the group classification of the phylogenetic analysis (Fig. 4A) is not necessarily reflected in the genome organization of *reb* genes. Thus, although the genome organization of the *reb* clusters of *Hy. taeniospiralis* and *Mm. mediterranea* are very similar to each other and cluster into Group 1, they are quite distinct from the “related” *reb* clusters of *Ro. alexandrii* and *Ps. chlororaphis*, and *Cb. taeniospiralis* and *Cm. varicaedens*, respectively, which are phylogenetically scattered. The *reb* cluster of *Ac. avenae* appears to be uniquely distinctive, both in the phylogenetic outline as well as in the *reb* cluster organization.

Finally, there are a number of genes present in the same genomic regions associated with *reb* gene clusters that do not share any homology with them (Fig. 4A, light gray). Raymann et al. (21) have previously classified some of these as “HP1.1” and “HP2.2” to “HP2.4” of the “Group 1 and 2 synteny patterns.” However, so far, there is no evidence that they have a structural role in R-body formation.

In summary, both our phylogenetic outline and the overall *reb* operon genomic organization point to four distinct functional Reb protein families, which are formed by a mosaic of species-dependent Reb proteins.

## DISCUSSION

In our study, we have shown that the various R-body morphological forms originally summarized in the pioneering review of Pond et al. (1) are not determined by a diagnostic gene organization signature and are widely distributed throughout Pseudomonadota (18, 19, 44–46).

The correlation of R-body morphology and kinetics with genetic information is not trivial. Although a relatively recent study employed genetic data to summarize phylogenetic relationships between species (21), the comparative biology of R-bodies is still unsatisfactory, since the phylogenetic studies have placed a large emphasis upon the well-studied *rebABDC* cluster of *Cb. taeniospiralis*, which is also experimentally accessible. However, as discussed below, the sequence homologies between “homologs” of different clusters are not maintained, implying that the ctRebABDC may not be optimal as a paradigm for R-bodies in general.

In this study, we have examined the question of whether the phylogenetic relationships between R-body genes, as well as the transcriptional organization, bear any relation to the observed morphologies. We have therefore focused our phylogenetic outline on six bacterial species for which R-bodies can be obtained experimentally and for which the chromosomal *reb* gene organization is also known. In our morphological studies, we have confirmed most of the observations of Pond et al. (1) and also extended the data employing high-quality images using a modern high-voltage TEM to image rolled-up isolated R-bodies and employing modern cryo-fixation methods to preserve cellular structures on ultra-thin sections. Finally, as a further comparative parameter, we examine the dynamic behavior of R-body unrolling, which offers a new functional parameter for R-body classification.

Our study confirms that the classification system reviewed by Pond et al. (1) is still useful, although it may require some extension since “intermediate” variants have also now been visualized. We also noted a morphological feature, hitherto undocumented, that rolled-up R-bodies of *Ac. avenae* and *Ma. mediterranea* show a “Pan flute” type geometry, where the layers of the R-body become successively longer as they approach the center. Of the additional morphological parameters summarized in Table 1, the parameter “repeat length per turn” is perhaps the most intriguing. Bearing in mind that the micrometer-scale R-bodies are assembled from nanometer-scale proteins, we can safely assume that the macroscopic unrolling morphology probably arises as the sum of concerted microscopic “sliding” events of the individual proteins of the R-body sheet. Now, if this sliding event were highly conserved at the molecular level, then we would expect the repeat length per turn to be independent of the length of the extended R-body. However, the repeat length per turn shows an extended length dependence that can be well fitted by a second-order polynomial (not shown). When molecular structures finally become available, it will be interesting to see how this macroscopic effect translates to a molecular microscopic event.

**TABLE 1:** Summary of the diagnostic morphological characteristics of R-bodies in various bacterial species^*a*^

Organism	Rolled-up diameter (µm)	Rolled-up height (µm)	Extended length (µm)	Repeat length per turn (mm)	Lamella thickness (nm)	Outer end	Inner end	Mode of unrolling	Sheath
*Cm. varicaedens* (α, ES, 7)	0.42 ± 0.11	0.43 ± 0.15	8.21 ± 2.36	1.04 ± 0.42	7.2 ± 0.7	b	t	i-u	y
*Ro. alexandrii* (α, NES, 51)	0.26 ± 0.05	0.27 ± 0.07	4.69 ± 0.98	0.65 ± 0.12	7.1 ± 0.4	t	t	i-u	n
*Ps. chlororaphis* (γ, NES, 51)	0.36 ± 0.08	0.50 ± 0.20	5.95 ± 1.76	0.85 ± 0.19	13.1 ± 0.7	t	t	i-u	n
*Ac. avenae* (β, NES, Pa)	0.34 ± 0.07	0.42 ± 0.07	4.62 ± 2.15	0.88 ± 0.17	11.9 ± 2.1	t	t	o-u	n
*Hy. taeniospiralis* (β, NES, Pt)	0.28 ± 0.06	0.30 ± 0.06	3.73 ± 0.85	0.80 ± 0.12	7.2 ± 1.4	b	t	i-u	y
*Ma. mediterranea* (γ, NES, 51)	0.26 ± 0.07	0.18 ± 0.03	4.25 ± 0.53	0.48 ± 0.07	7.7 ± 1.0	t	t	i-u	n

^*a*^ Based on transmission electron microscopy images of isolated R-bodies and R-bodies in high-pressure frozen bacterial cells, diameter, height, and lamella thickness were determined for rolled-up R-bodies. All parameters were calculated from 3 to 27 independent measurements each, depending on the species and parameter. Symbols in parentheses: α, β, and γ, proteobacterial class; ES and NES, endosymbiotic and non-endosymbiotic, respectively; 7, 51, Pa, and Pt, R-body type. b, blunt; t, tapered; y, yes; n, no.

Our optical dynamic analysis of R-body reversible unrolling showed that all isolates exhibited acid-induced unrolling, which, *in vivo*, is thought to occur through acidification (up to pH 3, see reference 47), thereby piercing both the host lysosomal/food vacuolar membrane, causing acid-induced cell lysis and resulting in the release of cytoplasmic contents to the external medium (cell killing). The physiological role of an alkali-induced R-body extension, observed here for the first time and unique to the R-bodies from *Cm. varicaedens* so far, is unclear. Paradoxically, the alkaline-induced extension accelerates the R-body full-length extension time to 100 ms, so that the kinetic profile is then essentially superimposable upon those of acid-unrolled R-bodies from *Ac. avenae* and *Ps. chlororaphis*. Another indication of the unique nature of the R-bodies is the SDS-induced unrolling behavior. We have obtained physical evidence (not shown here) that SDS shows strong binding to the *Cm. varicaedens* R-bodies, thereby modifying their physical properties. This is not generally true for the other R-body isolates in this work and may be due to a unique amino acid sequence in at least one of the cvReb proteins.

In our phylogenetic outline of R-body proteins with known morphologies, many of the Reb proteins separate into four reasonably well-separated regions (Fig. 4A, Groups 1–4), which also appear to contain well-defined subregions (see Group 1 in Fig. 4). However, this separation is not strict between species; e.g., the raReb and pcReb proteins are found in both Groups 2 and 3, and even the “paradigm” ctReb proteins do not fall into the same group. In contrast, the mmReb and htReb proteins, which show a somewhat distinctive gene organization (Fig. 4B) with respect to the other organisms, are indeed clustered within a single branch (Group 1). However, despite their genetic similarity, their R-body morphology is not identical (see Fig. 3 and 4).

We recognize that the construction of a reliable phylogenetic tree of Reb-homologous sequences with consistently high bootstrap values is currently not feasible with the current database, as Reb-homologous sequences can be highly diverse. However, a preliminary phylogenetic tree is still valuable, as it groups more closely homologous sequences and, as such, can show general genetic diversity and spread of sequences.

The detailed comparison of the Reb proteins from *Cb. taeniospiralis* and *Cm. varicaedens* reveals another level of complexity. It has been well established that for the “paradigm” ctReb proteins, ctRebA and ctRebB, which show strong homology to each other, form the “structural building block” (27) (which, in the absence of molecular structures, is still loosely defined). However, in *reb*-cluster 1 of *Cm. varicaedens*, cvRebA is only weakly homologous to cvRebB but is strongly homologous to cvRebC. The same is true for the cvRebA1/cvRebB1/cvRebC1 proteins of *cvReb* cluster 2. Thus, it seems likely that cvRebA/cvRebC are possible functional homologs of ctRebB. This would support the observation that the morphologies of the R-bodies from the two organisms (Type 7 [*Cb. taeniospiralis*] and Type 51 [*Cm. varicaedens*], respectively), as well as the unrolling dynamics (see below), are distinctly different ([1, 30], and this study). This probably also explains why the use of different “seed” Reb protein sequences can often yield quite different results in a phylogenetic analysis. Whether multiple operons occurring in the same species lead to hybrid or distinct R-bodies remains to be resolved.

Taken together, our data suggest that R-bodies from different bacterial species show no recognizable correlation between their presence in a particular class of Pseudomonadota, intracellular (ES) or extracellular (NES) localization, or an R-body classification type (Types 7, 51, Pa, and Pt). As such, they are probably an excellent paradigm for extensive lateral gene transfer between non-related (so far gram-negative) bacterial species (20, 21, 28).

It is tempting to suggest that this lateral gene transfer is related to the presence of the phage-like particles, which are often observed to coexist in some species of R-body-containing cells (see Fig. S2D and references 7, 41, 48), although no direct experimental evidence exists so far to confirm this hypothesis. Nevertheless, the distinct genetic organizations of some (unrelated) species (e.g., *Ro. alexandrii* and *Ps. chlororaphis*, or *Hy. taeniospiralis* and *Ma. mediterranea*, respectively) suggest that distinct functional R-body types may exist. This is supported by our optical analysis of R-body acid-induced unrolling kinetics *in vitro*, where various R-bodies can be classified into “fast burst” (100 ms extension time) (e.g., *Ps. chlororaphis* and *Ac. avenae*) and “slow” (about 0.8–3.0 s extension time) (e.g., *Cm. varicaedens* and *Ro. alexandrii*) extension types. When atomic structures finally become available, it will be intriguing to see whether the different extension kinetic variants are related to a defined and genetically recognizable structural motif. Ultimately, this knowledge will open new avenues for molecular design applications for these nanoscale thermal energy machines.

## MATERIALS AND METHODS

### Growth of strains and culture medium

*Paramecium caudatum* strain 224, harboring *Cm. varicaedens* endosymbionts in their macronucleus, was cultured in Erlenmeyer flasks. Strain 224 was originally gathered from the lake of the botanical garden at the University of Münster. Paramecia were grown in cerophyll straw infusion medium (49) at 23°C–25°C and fed twice or thrice a week with *Klebsiella* sp. growing in the same medium and at an identical temperature.

Non-endosymbiotic bacteria were received from the German Collection of Microorganisms and Cell Cultures GmbH (DSMZ). *Ps. chlororaphis* DSM 50083, *Ac. avenae* 7227, and *Hy. taeniospiralis* 2K1 were cultured in DSMZ Medium 1 (http://www.dsmz.de), *Ro. alexandrii* DFL-11 was cultured in DSMZ Medium 514 (Bacto Marine Broth), and *Ma. mediterranea* 23531 in DSMZ Medium 514f (Bacto Marine Broth). All NES strains were cultured in liquid media in Erlenmeyer flasks with or without shaking at 125 rpm or on sealed agar plates at 25°C or 26°C unless otherwise specified. Fresh medium was added to non-endosymbiotic bacteria in liquid media twice a week.

### Isolation of R-bodies

*Cm. varicaedens* R-bodies were isolated from *P. caudatum* cultures based on the protocol established by Bedingfield et al. (40). R-bodies of free-living bacteria were isolated either from liquid cultures or agar plates using methods for inclusion body isolation that were optimized in this study (50). Due to the widely varying amounts of R-bodies available from the ES and NES strains studied here, we employed four different isolation procedures (for full details see supplemental methods) as appropriate.

#### Large-scale isolation of R-bodies from *Cm. varicaedens*

A volume of 20–40 L *P. caudatum* culture was starved for 9–11 days before isolation. The culture was filtered through gauze (eight layers), concentrated using a dairy centrifuge, and then subsequently gauze-filtered again. The filtrate was centrifuged for 3 min at 300 × *g*, and the pellet was homogenized using a glass homogenizer in Buffer A (50 mM TrisHCl, pH 8.5), then subjected to four freeze-thaw cycles, followed by incubation in Buffer A containing 2% (wt/vol) OPOE (Bachem, Switzerland) at 4°C for 48 h. Finally, the detergent-extracted R-bodies were washed in Buffer A and then suspended in a small volume (usually about 2 mL) of the same buffer. The resuspended R-body preparation was then stored at 4°C or flash-frozen in liquid nitrogen prior to storage at −80°C.

#### Medium-scale (400 mL) isolation of R-bodies

This method was used for cultures of *Ac. avenae*, *Ma. mediterranea*, and *Hy. taeniospiralis*. The bacteria were grown in their respective media (see above) and, if appropriate (*Ac. avenae* and *Ma. mediterranea*), starved for 6–8 days before isolation, then harvested by centrifugation. The resulting pellets were suspended in 7.5 mL Buffer A and then subjected to six freeze-thaw cycles for initial cell breakage, followed by the addition of hen egg white lysozyme (HEWL, 0.13 mg/mL, Sigma-Aldrich GmbH) and EDTA (33 mM), incubation at 37°C for 1 h and then overnight at room temperature. The lysed cells were then subjected to five further freeze-thaw cycles, and the chromosomal DNA released after breakage was digested by the addition of DNAase I (9 µg/mL) and MgCl_2_ (8 mM) at 37°C for 30 min, followed by overnight incubation at room temperature. The breakage lysates were solubilized for 3 days at room temperature in the presence of 1% (vol/vol) Triton X-100/1% (wt/vol) SDS, then centrifuged to obtain a pellet, which was washed twice using Buffer A and then purified by centrifugation at 64 × *g* (*Ac. avenae*) or 240 × *g* (*Ma. mediterranea*). The supernatant was then centrifuged at 20,800 × *g* to obtain the R-bodies as a pellet and then suspended in 1 mL of Buffer A prior to storage as described in “Large-scale isolation of R-bodies from *Cm. varicaedens*.”

#### Small-scale isolation of R-bodies from 40 mL cultures

This method was used for *Hy. taeniospiralis* and *Ma. mediterranea*, respectively, and was performed using a procedure similar to that described in “Medium-scale (400 mL) isolation of R-bodies,” except that Buffer A was replaced with 50 mM TrisHCl (pH 8.5) containing 100 mM NaCl and 1 mM EDTA (Buffer B), only four freeze-thaw cycles were performed, and the amounts of HEWL and DNAase I were modified (0.3 mg/mL and 8 µg/mL, respectively). In addition, the long incubation steps at room temperature were omitted, and the detergent solubilization employed only Triton X-100 (1.2% [vol/vol] and 0.9% [vol/vol] for *Hy. taeniospiralis* and *Ma. mediterranea*, respectively), with an overnight incubation at 4°C. R-bodies were then washed and harvested by centrifugation as described in “Medium-scale (400 mL) isolation of R-bodies.”

#### Very small-scale isolation of R-bodies from agar plate cultures

This method was used for *Ro. alexandrii*, *Ac. avenae*, and *Ps. chlororaphis*. Two agar plate cultures were scraped up and suspended in either Buffer B (*Ro. alexandrii* and *Ac. avenae*) or 1 mL medium (*Ps. chlororaphis*). For *Ro. alexandrii* and *Ac. avenae,* the plates were illuminated with UV-A light (5 W, 365 nm at about 8 cm for 90 s) and then incubated overnight at 25°C–26°C before harvesting. The pooled suspensions were subjected to four freeze-thaw cycles and then incubated with 1.6 mg/mL HEWL for 60 min at 37°C. The pools were subjected to an initial detergent solubilization step (0.5%–0.65% [vol/vol] Triton X-100) for 1 h at 37°C. The partially solubilized lysates of *Ro. alexandrii* and *Ps. chlororaphis* were fragmented further by ultrasonication using an ultrasonication tip (Bandelin Sonopuls Homogeniser UW3100, Germany) for 10 min using a 5 s duty cycle or an ultrasonic bath for 2 h using a 10 min duty cycle, respectively. After subsequent incubation with DNAase I (8 µg/mL) in Buffer B containing 7.5 mM MgCl_2_ (10 mM CaCl_2_ was also added to the lysate from *Ac. avenae*), the lysates were pelleted by centrifugation, resuspended in Buffer A, and then subjected to a second detergent solubilization step using either 0.65% (vol/vol) (*Ro. alexandrii*), 1.2% (vol/vol) (*Ac. avenae*), or 0.5% (vol/vol) (*Ps. chlororaphis*) Triton X-100. Finally, the pellets were washed twice by centrifugation using Buffer A for each resuspension step. Isolated R-bodies of *Ro. alexandrii* were purified with a further centrifugation step at 68 × *g* as described in “Medium-scale (400 mL) isolation of R-bodies.” The final pellet was resuspended in about 1 mL Buffer A and stored as described in “Large-scale isolation of R-bodies from *Cm. varicaedens*.”

### Preparation for electron microscopy

Isolated R-bodies were prepared for electron microscopy (EM) using standard negative contrast imaging with 1% (wt/vol) phosphotungstic acid. If required, R-bodies in the isolate were unrolled by dilution with an equal volume of 1 M HCl before adsorption to the grid.

For the preparation of whole bacteria or *Paramecium* cells for EM, R-body production was induced, and NES cells and paramecia were harvested by centrifugation. Paramecia were additionally suspended in ~10% (wt/vol) dextran. Cells were vitrified using a high-pressure freezer (Leica ICE) employing either two gold carriers for paramecia or aluminum carriers for bacteria. Frozen samples (1.0–1.5 mL each) were freeze-substituted using a Leica/Reichert AFS apparatus and then embedded in Spurr resin using standard procedures (51).

The resin-embedded samples were thin sectioned into 60–70 nm thick slices with an ultramicrotome (Leica Ultracut UCT) (52). Full details are given in the supplemental methods.

### Electron microscopy

Transmission electron microscopy of samples was performed either at 60 kV using the EM10A (Carl Zeiss) electron microscope or at 300 kV using the Spectra 300 electron microscope (ThermoFisher Scientific). Images were taken using either a TRS Slowscan CCD 1K camera (Tröndle Restlichtverstärkersysteme) for the EM10A or a Ceta 4K camera for the Spectra 300. All images were stored in 8-bit TIFF format.

### Light microscopy of isolated R-bodies

Shape-transitioning of isolated R-bodies for various environmental unrolling triggers (pH and detergent) was observed by phase-contrast microscopy with an Axiovert 200M (Carl Zeiss) inverted microscope employing a Plan-Neofluar 40×/1.30 oil Ph3 objective.

R-body samples (2.5–5 µL) were pipetted onto a large cover slip serving as the glass slide and covered with a smaller cover slip. The cover slip assembly was then placed in the microscope, then the sample was washed with Milli-Q water (1–3× the sample volume). After letting the solution equilibrate for up to 30 s, the kinetic transition was induced by the addition of a reagent (equal in volume to the samples). The reagents were either 1 M HCl, 0.5 M NaOH, or 10% (wt/vol) SDS (see Fig. 3). Images were captured in RAW format and videos in MP4 format (IPB compression) using a Canon EOS R7 camera. Videos were either recorded in 4K or 4K Crop at 60 fps or in full HD at 120 fps.

The shape-transition speed of R-bodies was determined by measuring the time-dependent distance between R-body endpoints, as determined frame-by-frame using ImageJ. The shape transition was always measured from the last frame before a change in the R-body appearance became visible. R-bodies that appeared to arrest for longer periods of time (≥0.5  s) were excluded. At least nine extension events were averaged per species for each environmental trigger (HCl, NaOH, and SDS).

The pH sensitivity of isolated R-bodies was tested by pelleting isolated rolled-up R-bodies in a tabletop centrifuge at 20,800 × *g* for 10 min and then resuspending them in solutions adjusted to different pH values. Either HCl (0.1 M, pH 1.0; 0.01 M, pH 2.0), 0.1 M citrate buffer (pH 3.0, 4.0, 5.0, and 6.0, respectively), or ROTIStar buffer (Carl Roth GmbH) (pH 7.0) was used to adjust the solution to the desired pH. To determine the pH at which R-bodies retract to their initial state, unrolled R-bodies were pelleted as described above and resuspended in either 0.1 M carbonate-bicarbonate buffer (pH 10.0 or 10.8) or NaOH (0.01 M, pH 12.0; 0.1 M, pH 13.0; and 1 M, pH 14.0). R-bodies were then checked for their extended/retracted state by phase-contrast microscopy using an AxioScope.A1 (Carl Zeiss) light microscope fitted with an N-Achroplan 40×/0.65 Ph2 or an EC Plan-Neofluar 63×/1.25 oil Ph3 objective, respectively.

### Phylogenetic analysis

Genome data from 56 bacterial strains with RebB homologs (271 sequences in total) were obtained from the present NCBI database. For the selection of species and strains, the data sets used by Raymann et al. (21) and Wang et al. (20) were used as a basis. Sequences of strains not classified down to species level and where less than two Reb homologs were found were removed. The resulting data set was then supplemented with all Reb homologous sequences of strains experimentally proven to be able to produce R-bodies. Reb homologous sequences of additional species (e.g., *Ca*. Bealeia paramacronuclearis or *Anabaena azotica*) were included that we found using a BlastP search (53–55) on sequences of species that lacked homologous sequences in previous data sets (e.g., for *Caedimonas varicaedens* or *Acidovorax avenae*). Furthermore, we included Reb homologous sequences of species belonging to flavo- and cyanobacteria (e.g., *Chryseobacterium indologenes* or *Aphanothece sacrum*) that we identified with BlastP (53–55). For *Cm. varicaedens*, the database draft annotation from the published *reb* cluster lacks the 5′-end from *rebA*. We therefore isolated the complete *reb* cluster 1 by PCR and re-sequenced the *rebABCD* region (see below). These data replaced the corresponding sequences on the NCBI database in the phylogenetic analysis below. The NCBI genome accession numbers for the seven species used here are as follows: *Cb. taeniospiralis*, ASM280329v2; *Cm. varicaedens*, ASM119265v1; *Ro. alexandrii*, ASM15809v2; *Ps. chlororaphis*, ASM394576v1; *Ac. avenae*, ASM17685v2; *Hy. taeniospiralis*, ASM159230v1; and *Ma. mediterranea*, ASM19286v1.

Amino acid sequences of genes annotated as RebB homologs, non-annotated RebB homologs (such as those for *Ac. avenae*), as well as surrounding genes, were extracted, and an initial phylogenetic UPGMA tree was created based on sequence alignment of 676 sequences with MAFFT version 7 (56, 57). Based on this first tree, sequences without strong relation to Reb proteins were excluded, a second alignment was performed for 424 sequences, and a maximum-likelihood tree was generated with the IQ-Tree webserver version 1.6.12 (58, 59). Model LG with empirical base frequencies and gamma 4 was used as determined by ModelFinder (60), and an ultrafast bootstrap analysis (61) was performed with 1,000 replicates. The tree was then further refined to focus only on potential structural Reb proteins (e.g., “hypothetical proteins” of the Group 1 and 2 synteny patterns were excluded, see Raymann et al. [21]). A final alignment of 270 sequences was then performed in MAFFT with strategy L-INS-i, which is recommended for sequences with one conserved domain, as is commonly observed for Reb proteins. The final maximum-likelihood tree was then generated as described above. For visualization, the tree was rendered with ggplot2 (62) with the programming language R (63) and manually annotated using Inkscape. For the rootless equal angle visualization (Fig. 4A), plpRebB (see Table S1) was defined as the root for visual purposes. For the rectangular tree (Fig. S1), the sequence of cvRebE was added to the final alignment and then used as an outgroup. Sequences were grouped based on relation within the tree.

### Re-sequencing of the *reb* operon in *Cm. varicaedens*

**Fig 5:**
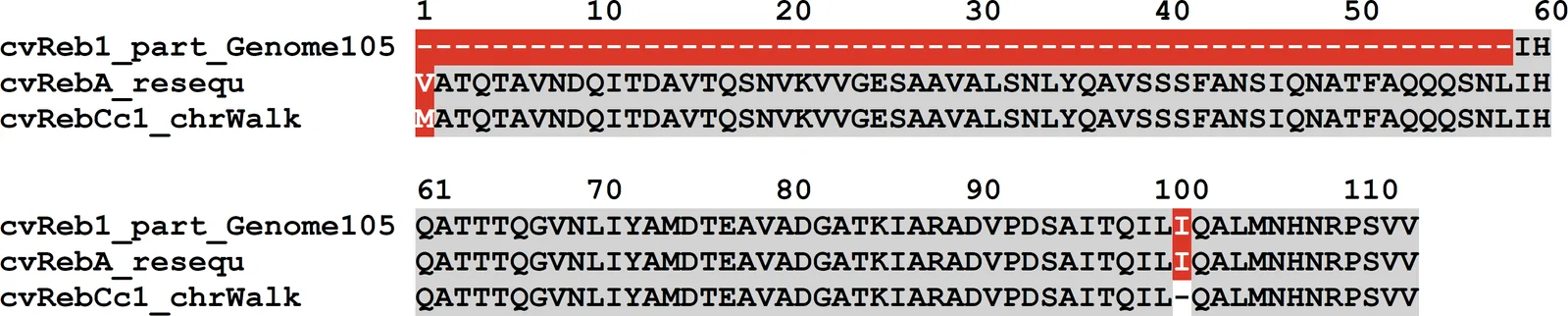
Comparison of cvRebA sequences from *Cm. varicaedens* as obtained by different methods: Genome105, the genomic sequence present on Cva_contig000105 (accession no.: BBVC01000105); resequ, the complete cvRebA sequence present on a PCR-derived fragment obtained from chromosomal DNA of *Cm. varicaedens*; and chrwalk, initial cvRebA sequence, obtained by a chromosomal walking technique (56).

The *rebABCD* operon from *Cm. varicaedens* was initially sequenced by Schrallhammer (64) using a primer-driven chromosomal walking. More recently, the *reb* operon was also annotated in a draft genome sequence (65) of *Cm. varicaedens* deposited in the NCBI database (accession no. BBVC01000105), but in this draft shotgun sequence, the 5′-region of *rebA* was lacking. We therefore re-checked the *rebABCD* sequence by isolating a *rebABCD* fragment by PCR using primers derived from both of the above sequences. For PCR, the following primers, derived from the Schrallhammer (64) sequence data, were used: RebCv1F and RebCv4R (Table 2), and the initial template DNA, the total chromosomal DNA (approximately 0.1 ng), was isolated from *Cm. varicaedens* using a standard protocol (alkaline cell lysis, followed by phenol/chloroform washing, and then precipitation with ethanol/3 M Na-acetate). The PCR cycle using DreamTaq Hot Start DNA polymerase (ThermoFisher Scientific) was started at an initial denaturation (95°C for 1 min), followed by denaturation (95°C for 30 s), annealing (53.8°C–60.2°C, 30 s temperature gradient), and elongation (72°C for 1 min) steps. A final 5 min elongation step was included at the end of the PCR run. The PCR-derived fragment was visualized and isolated using agarose (1.5%) gel electrophoresis and then cloned into the vector pGEM-T-easy (Promega Corporation) to obtain the vector pGMRebABCD. The pGEM insert was then sequenced commercially using standard pUC universal and reverse primers to confirm the *rebABCD* fragment. Complete sequence coverage in both directions was obtained using the additional primers listed in Table 2. A comparison of the RebA sequences from the three sources is shown in Fig. 5.

**TABLE 2:** Primers derived from the sequence data in reference 64 used for gradient PCR for the re-sequencing of *rebABCD*

Primer	Sequence
RebCv1F	5′ CAAGCCCCCTGGGGGATG 3′
RebCv1R	5′ AGAAGGACGATTATGATTCAT C 3′
RebCv2F	5′ GAATCATAATCG TCCTTCTGTTG 3′
RebCv3R	5′ GCTGCCTTTTTCAATGCCGC 3′
RebCv4F	5′ GCGGCATTGAAAAAGGCAGC 3′
RebCv4R	5′ CATTACGCGTCGAGCAGTCCCAAAATC 3′

## Data Availability

A full list of the protein sequences used for the phylogenetic analysis, including their respective NCBI protein IDs, is given in the supplemental material. The results of the re-sequencing of the *Cm. varicaedens reb* clusters have been deposited at DaRUS, the data repository of the University of Stuttgart, and are publicly accessible at https://doi.org/10.18419/DARUS-6419. Detailed protocols for R-body isolation from ES and NES species and electron microscopic methods, as well as videos of the experiments summarized in Fig. 3, are included in the supplemental material.

## SUPPLEMENTAL MATERIAL

Supplemental material aem.00864-26-s0001.docx Fig. S1 and S2; supplemental legends and methods.

Table S1 aem.00864-26-s0002.pdf Complete list of protein sequences used for the phylogenetic outline.

Video S1A aem.00864-26-s0003.mp4 Light microscopic video recording of acid-induced unrolling of an R-body isolated from *Caedimonas varicaedens*.

Video S1B aem.00864-26-s0004.mp4 Light microscopic video recording of alkali-induced unrolling of an R-body isolated from *Cm. varicaedens*.

Video S1C aem.00864-26-s0005.mp4 Light microscopic video recording of alkali-induced unrolling of an R-body isolated from *Cm. varicaedens*.

Video S1D aem.00864-26-s0006.mp4 Light microscopic video recording of acid-induced unrolling of an R-body isolated from *Ro. alexandrii*.

Video S1E aem.00864-26-s0007.mp4 Light microscopic video recording of acid-induced unrolling of an R-body isolated from *Ps. chlororaphis*.

Video S1F aem.00864-26-s0008.mp4 Light microscopic video recording of acid-induced unrolling of an R-body isolated from *Ac. avenae.*

ASM does not own the copyrights to Supplemental Material that may be linked to, or accessed through, an article. The authors have granted ASM a non-exclusive, world-wide license to publish the Supplemental Material files. Please contact the corresponding author directly for reuse.
