# Supplementary material for: Bacterial R-bodies with common morphologies and unrolling dynamics are phylogenetically scattered, indicating extensive lateral gene transfer and wide application potential
Source: Appl Environ Microbiol. 2026 Sep 11;92(10):e00864-26. doi: 10.1128/aem.00864-26 (PMC13689365; doi:10.1128/aem.00864-26)
Supplement: Supplementary file 1 [file aem.00864-26-s0002.pdf]

#	Class	Species	Abbr.	Strain	NCBI RefSeq	Protein ID	NCBI Protein ID	Group	Length
1	α-PB	*Azorhizobium caulinodans*	azc	ORS 571	NC_009937.1	azcA	WP_012172304.1	3	74
2	α-PB	*Azorhizobium caulinodans*	azc	ORS 571	NC_009937.1	azcB	WP_043879617.1	ungrouped	96
3	α-PB	*Azorhizobium caulinodans*	azc	ORS 571	NC_009937.1	azcC	WP_012172306.1	3	113
4	α-PB	*Azorhizobium caulinodans*	azc	ORS 571	NC_009937.1	azcE	WP_043879618.1	ungrouped	119
5	α-PB	*Azospirillum baldaniorum*	azb	Sp245	NZ_CP022262	azbA	WP_014200159.1	1	133
6	α-PB	*Azospirillum baldaniorum*	azb	Sp245	NZ_CP022262	azbB	WP_014200160.1	1	74
7	α-PB	*Azospirillum baldaniorum*	azb	Sp245	NZ_CP022262	azbC	WP_014200161.1	1	129
8	α-PB	*Azospirillum baldaniorum*	azb	Sp245	NZ_CP022262	azbD	WP_014200162.1	1	116
9	α-PB	*Ca. Bealeia paramacronuclearis*	bep	US_Bl 11III1	JAVHWZ010000006	bepA	MEB3703329.1	1B	111
10	α-PB	*Ca. Bealeia paramacronuclearis*	bep	US_Bl 11III1	JAVHWZ010000006	bepB	MEB3703328.1	1B	105
11	α-PB	*Ca. Bealeia paramacronuclearis*	bep	US_Bl 11III1	JAVHWZ010000006	bepC	MEB3703327.1	1B	74
12	α-PB	*Ca. Bealeia paramacronuclearis*	bep	US_Bl 11III1	JAVHWZ010000006	bepD	MEB3703325.1	1B	98
13	α-PB	*Ca. Bealeia paramacronuclearis*	bep	US_Bl 11III1	JAVHWZ010000006	bepE	MEB3703324.1	3	116
14	α-PB	*Ca. Bealeia paramacronuclearis*	bep	US_Bl 11III1	JAVHWZ010000014	bepA1	MEB3703404.1	1B	106
15	α-PB	*Ca. Bealeia paramacronuclearis*	bep	US_Bl 11III1	JAVHWZ010000014	bepB1	MEB3703405.1	1B	111
16	α-PB	*Ca. Bealeia paramacronuclearis*	bep	US_Bl 11III1	JAVHWZ010000016	bepA2	MEB3703408.1	3	103
17	α-PB	*Ca. Finniella inopinata*	fii	HOLO01	NZ_SCFB01000004	fiiA	WP_130153720.1	1B	77
18	α-PB	*Ca. Finniella inopinata*	fii	HOLO01	NZ_SCFB01000004	fiiB	WP_130153719.1	3	106
19	α-PB	*Ca. Finniella inopinata*	fii	HOLO01	NZ_SCFB01000004	fiiC	WP_130153718.1	1B	111
**20**	**α-PB**	***Caedimonas varicaedens***	**cv**	-	-	**cvA**	-	1B	**113**
**21**	**α-PB**	***Caedimonas varicaedens***	**cv**	-	-	**cvB**	-	ungrouped	**108**
**22**	**α-PB**	***Caedimonas varicaedens***	**cv**	-	-	**cvC**	-	1B	**115**
**23**	**α-PB**	***Caedimonas varicaedens***	**cv**	-	-	**cvD**	-	3	**71**
**24**	**α-PB**	***Caedimonas varicaedens***	**cv**	-	-	**cvE**	-	other	**76**
**25**	**α-PB**	***Caedimonas varicaedens***	**cv**	-	**NZ_BBVC01000009**	**cvA1**	**WP_075262328.1**	1B	**109**
**26**	**α-PB**	***Caedimonas varicaedens***	**cv**	-	**NZ_BBVC01000009**	**cvB1**	**WP_062139070.1**	ungrouped	**122**
**27**	**α-PB**	***Caedimonas varicaedens***	**cv**	-	**NZ_BBVC01000009**	**cvC1**	**WP_062139068.1**	1B	**74**
**28**	**α-PB**	***Caedimonas varicaedens***	**cv**	-	**NZ_BBVC01000009**	**cvD1**	**WP_062139066.1**	3	**109**
**29**	**α-PB**	***Caedimonas varicaedens***	**cv**	-	**NZ_BBVC01000027**	**cvA2**	**WP_062139929.1**	1B	**104**
30	α-PB	*Eilatimonas milleporae*	eim	DSM 25217	NZ_REFR01000009	eimB	WP_211332078.1	ungrouped	78
31	α-PB	*Eilatimonas milleporae*	eim	DSM 25217	NZ_REFR01000009	eimC	WP_121937581.1	ungrouped	108
32	α-PB	*Eilatimonas milleporae*	eim	DSM 25217	NZ_REFR01000009	eimD	WP_121937580.1	ungrouped	99
33	α-PB	*Eilatimonas milleporae*	eim	DSM 25217	NZ_REFR01000009	eimE	WP_121937579.1	ungrouped	73
34	α-PB	*Eilatimonas milleporae*	eim	DSM 25217	NZ_REFR01000009	eimF	WP_121937578.1	ungrouped	91
35	α-PB	*Eilatimonas milleporae*	eim	DSM 25217	NZ_REFR01000009	eimG	WP_121937577.1	ungrouped	76
36	α-PB	*Kordiimonas lipolytica*	kol	M41	NZ_LRUB01000036	kolA	WP_068153267.1	ungrouped	91
37	α-PB	*Kordiimonas lipolytica*	kol	M41	NZ_LRUB01000036	kolB	WP_068153269.1	ungrouped	104
38	α-PB	*Kordiimonas lipolytica*	kol	M41	NZ_LRUB01000036	kolC	WP_068153272.1	ungrouped	116
39	α-PB	*Kordiimonas lipolytica*	kol	M41	NZ_LRUB01000036	kolD	WP_068153275.1	ungrouped	141
40	α-PB	*Kordiimonas lipolytica*	kol	M41	NZ_LRUB01000036	kolE	WP_068153278.1	ungrouped	107
41	α-PB	*Kordiimonas lipolytica*	kol	M41	NZ_LRUB01000036	kolF	WP_068153280.1	ungrouped	108
42	α-PB	*Kordiimonas lipolytica*	kol	M41	NZ_LRUB01000036	kolG	WP_068153284.1	3	109
43	α-PB	*Kordiimonas lipolytica*	kol	M41	NZ_LRUB01000036	kolH	WP_068153288.1	ungrouped	109
44	α-PB	*Kordiimonas lipolytica*	kol	M41	NZ_LRUB01000036	kolI	WP_068153294.1	3	108
45	α-PB	*Nisaea acidiphila*	nia	MEBiC11861	NZ_CP102480	niaA	WP_257766547.1	ungrouped	108
46	α-PB	*Nisaea acidiphila*	nia	MEBiC11861	NZ_CP102480	niaB	WP_257766548.1	ungrouped	109
47	α-PB	*Nisaea acidiphila*	nia	MEBiC11861	NZ_CP102480	niaC	WP_257766549.1	ungrouped	101
48	α-PB	*Nisaea acidiphila*	nia	MEBiC11861	NZ_CP102480	niaD	WP_257766550.1	3	121
49	α-PB	*Nisaea acidiphila*	nia	MEBiC11861	NZ_CP102480	niaE	WP_257766551.1	ungrouped	76
50	α-PB	*Nisaea acidiphila*	nia	MEBiC11861	NZ_CP102480	niaF	WP_257766552.1	3	108
51	α-PB	*Oceanibacterium hippocampi*	och	CECT 7691	NZ_FWFR01000002	ochB	WP_085883884.1	ungrouped	76
52	α-PB	*Oceanibacterium hippocampi*	och	CECT 7691	NZ_FWFR01000002	ochC	WP_085883885.1	ungrouped	76
53	α-PB	*Oceanibacterium hippocampi*	och	CECT 7691	NZ_FWFR01000002	ochD	WP_085883886.1	ungrouped	109
54	α-PB	*Oceanibacterium hippocampi*	och	CECT 7691	NZ_FWFR01000002	ochE	WP_085883887.1	ungrouped	101
55	α-PB	*Oceanibacterium hippocampi*	och	CECT 7691	NZ_FWFR01000002	ochF	WP_085883888.1	ungrouped	99
56	α-PB	*Oceanicaulis alexandrii*	oca	DSM 11625	NZ_ATUP01000001	ocaA	WP_022700372.1	3	107
57	α-PB	*Oceanicaulis alexandrii*	oca	DSM 11625	NZ_ATUP01000001	ocaC	WP_022700367.1	ungrouped	114
58	α-PB	*Polymorphum gilvum*	pog	SL003B-26A1	NC_015259	pogA	WP_013652168.1	3	96
59	α-PB	*Polymorphum gilvum*	pog	SL003B-26A1	NC_015259	pogC	WP_013652173.1	2	89

60	α-PB	*Polymorphum gilvum*	pog	SL003B-26A1	NC_015259	pogD	WP_013652174.1	ungrouped	88
61	α-PB	*Rhodocista centenaria*	rhc	SW	NC_011420.2	rhcA	WP_012567179.1	ungrouped	90
62	α-PB	*Rhodocista centenaria*	rhc	SW	NC_011420.2	rhcC	WP_012567177.1	ungrouped	132
63	α-PB	*Rhodocista centenaria*	rhc	SW	NC_011420.2	rhcD	WP_012567176.1	ungrouped	115
64	α-PB	*Rhodocista centenaria*	rhc	SW	NC_011420.2	rhcE	WP_012567175.1	ungrouped	117
65	α-PB	*Rhodocista centenaria*	rhc	SW	NC_011420.2	rhcF	WP_012567174.1	ungrouped	107
66	α-PB	*Rhodocista centenaria*	rhc	SW	NC_011420.2	rhcG	WP_234703853.1	ungrouped	108
**67**	**α-PB**	***Roseibium alexandrii***	**ra**	**DFL-11**	**NZ_CM011002.1**	**raA**	**WP_008188621.1**	3	**102**
**68**	**α-PB**	***Roseibium alexandrii***	**ra**	**DFL-11**	**NZ_CM011002.1**	**raC**	**WP_040450837.1**	2	**115**
**69**	**α-PB**	***Roseibium alexandrii***	**ra**	**DFL-11**	**NZ_CM011002.1**	**raD**	**WP_134852858.1**	ungrouped	**92**
70	α-PB	*Roseibium aquae*	roa	CGMCC 1.12426	NZ_VXDC01000001	roaA	WP_150494198.1	3	104
71	α-PB	*Roseibium aquae*	roa	CGMCC 1.12426	NZ_VXDC01000001	roaC	WP_150494208.1	2	105
72	α-PB	*Roseibium aquae*	roa	CGMCC 1.12426	NZ_VXDC01000001	roaD	WP_206668290.1	ungrouped	117
73	α-PB	*Roseibium limicola*	rol	CAU 1637	NZ_JAFLNF010000001	rolA	WP_206937144.1	3	61
74	α-PB	*Roseibium limicola*	rol	CAU 1637	NZ_JAFLNF010000001	rolC	WP_206937132.1	2	110
75	α-PB	*Roseibium limicola*	rol	CAU 1637	NZ_JAFLNF010000001	rolD	WP_206937130.1	ungrouped	110
76	α-PB	*Ruegeria pomeroyi*	rup	DSS-3	NC_006569	rupA	WP_011242170.1	3	116
77	α-PB	*Ruegeria pomeroyi*	rup	DSS-3	NC_006569	rupC	WP_011242175.1	2	75
78	α-PB	*Ruegeria pomeroyi*	rup	DSS-3	NC_006569	rupD	WP_011242176.1	2	106
79	α-PB	*Skermanella stibiiresistens*	sks	SB22	NZ_AVFL01000018	sksA	WP_037457066.1	ungrouped	81
80	α-PB	*Skermanella stibiiresistens*	sks	SB22	NZ_AVFL01000018	sksB	WP_037457063.1	ungrouped	107
81	α-PB	*Skermanella stibiiresistens*	sks	SB22	NZ_AVFL01000018	sksD	WP_037457061.1	ungrouped	111
82	α-PB	*Skermanella stibiiresistens*	sks	SB22	NZ_AVFL01000018	sksE	WP_037457060.1	ungrouped	99
83	α-PB	*Skermanella stibiiresistens*	sks	SB22	NZ_AVFL01000018	sksF	WP_037457057.1	ungrouped	101
84	α-PB	*Skermanella stibiiresistens*	sks	SB22	NZ_AVFL01000018	sksH	WP_157619477.1	ungrouped	75
85	α-PB	*Sneathiella aquimaris*	sna	216LB-ZA1-12	NZ_CP112881	snaB	WP_169545937.1	ungrouped	107
86	α-PB	*Sneathiella aquimaris*	sna	216LB-ZA1-12	NZ_CP112881	snaC	WP_169545938.1	ungrouped	87
87	α-PB	*Sneathiella aquimaris*	sna	216LB-ZA1-12	NZ_CP112881	snaD	WP_169545939.1	ungrouped	107
88	α-PB	*Sphingomonas kyeonggiensis*	spk	DSM 101806	NZ_JACIEH010000002	spkB	WP_183997450.1	1	107
89	α-PB	*Sphingomonas kyeonggiensis*	spk	DSM 101806	NZ_JACIEH010000002	spkC	WP_183997449.1	1	111
90	α-PB	*Thalassospira australica*	tha	NP 3b2	NZ_JRJE01000004	thaA	WP_033067647.1	ungrouped	98
91	α-PB	*Thalassospira australica*	tha	NP 3b2	NZ_JRJE01000004	thaB	WP_033067648.1	ungrouped	92
92	α-PB	*Thalassospira australica*	tha	NP 3b2	NZ_JRJE01000004	thaC	WP_033067649.1	ungrouped	74
93	α-PB	*Thalassospira australica*	tha	NP 3b2	NZ_JRJE01000004	thaD	WP_052065425.1	ungrouped	107
94	α-PB	*Thalassospira australica*	tha	NP 3b2	NZ_JRJE01000004	thaE	WP_033067651.1	3	86
95	α-PB	*Thalassospira australica*	tha	NP 3b2	NZ_JRJE01000004	thaF	WP_033067652.1	ungrouped	115
96	α-PB	*Thalassospira australica*	tha	NP 3b2	NZ_JRJE01000004	thaG	WP_033067653.1	3	107
97	α-PB	*Thalassospira profundimaris*	thp	WP0211	NZ_AMRN01000005	thpA	WP_008890488.1	ungrouped	116
98	α-PB	*Thalassospira profundimaris*	thp	WP0211	NZ_AMRN01000005	thpB	WP_008890487.1	ungrouped	108
99	α-PB	*Thalassospira profundimaris*	thp	WP0211	NZ_AMRN01000005	thpC	WP_008890486.1	ungrouped	165
100	α-PB	*Thalassospira profundimaris*	thp	WP0211	NZ_AMRN01000005	thpD	WP_008890485.1	ungrouped	115
101	α-PB	*Thalassospira profundimaris*	thp	WP0211	NZ_AMRN01000005	thpE	WP_008890484.1	ungrouped	106
102	α-PB	*Thalassospira profundimaris*	thp	WP0211	NZ_AMRN01000005	thpF	WP_008890482.1	3	118
103	α-PB	*Thalassospira profundimaris*	thp	WP0211	NZ_AMRN01000005	thpG	WP_008890481.1	ungrouped	92
104	α-PB	*Thalassospira profundimaris*	thp	WP0211	NZ_AMRN01000005	thpH	WP_008890480.1	3	89
**105**	**β-PB**	***Acidovorax avenae***	**aa**	**ATCC 19860**	**NC_015138.1**	**aaA**	**WP_013596745.1**	ungrouped	**110**
**106**	**β-PB**	***Acidovorax avenae***	**aa**	**ATCC 19860**	**NC_015138.1**	**aaB**	**WP_013596746.1**	ungrouped	**122**
**107**	**β-PB**	***Acidovorax avenae***	**aa**	**ATCC 19860**	**NC_015138.1**	**aaC**	**WP_013596747.1**	ungrouped	**107**
**108**	**β-PB**	***Acidovorax avenae***	**aa**	**ATCC 19860**	**NC_015138.1**	**aaD**	**WP_013596748.1**	ungrouped	**105**
109	β-PB	*Burkholderia ambifaria*	bua	AMMD	NZ_CP009798	buaA	WP_011656534.1	ungrouped	95
110	β-PB	*Burkholderia ambifaria*	bua	AMMD	NZ_CP009798	buaB	WP_011656535.1	ungrouped	89
111	β-PB	*Burkholderia ambifaria*	bua	AMMD	NZ_CP009798	buaC	WP_011656536.1	ungrouped	89
112	β-PB	*Burkholderia ambifaria*	bua	AMMD	NZ_CP009798	buaD	WP_011656539.1	3	89
113	β-PB	*Burkholderia glumae*	bug	BGR1	NC_012721	bugA	WP_015877694.1	1	90
114	β-PB	*Burkholderia glumae*	bug	BGR1	NC_012721	bugB	WP_015877693.1	1	90
115	β-PB	*Burkholderia glumae*	bug	BGR1	NC_012721	bugC	WP_015877692.1	1	96
116	β-PB	*Burkholderia glumae*	bug	BGR1	NC_012721	bugD	WP_015877691.1	1	89
117	β-PB	*Burkholderia glumae*	bug	BGR1	NC_012721	bugE	WP_015877690.1	1	88
118	β-PB	*Burkholderia glumae*	bug	BGR1	NC_012721	bugF	WP_015877689.1	1	90
119	β-PB	*Burkholderia oklahomensis*	buo	EO147	NZ_CP013357	buoA	WP_010101057.1	1	96

120	β-PB	*Burkholderia oklahomensis*	buo	EO147	NZ_CP013357	buoB	WP_010101056.1	1	89
121	β-PB	*Burkholderia oklahomensis*	buo	EO147	NZ_CP013357	buoC	WP_006024228.1	1	88
122	β-PB	*Burkholderia oklahomensis*	buo	EO147	NZ_CP013357	buoD	WP_010101053.1	1	90
123	β-PB	*Chromobacterium violaceum*	chv	ATCC 12472	NC_005085	chvA	WP_011134939.1	2	103
124	β-PB	*Chromobacterium violaceum*	chv	ATCC 12472	NC_005085	chvA1	WP_011134288.1	3	103
125	β-PB	*Chromobacterium violaceum*	chv	ATCC 12472	NC_005085	chvC1	WP_011134279.1	2	169
126	β-PB	*Chromobacterium violaceum*	chv	ATCC 12472	NC_005085	chvD1	WP_011134278.1	2	115
127	β-PB	*Chromobacterium violaceum*	chv	ATCC 12472	NC_005085	chvE1	WP_011134277.1	2	98
128	β-PB	*Chromobacterium violaceum*	chv	ATCC 12472	NC_005085	chvF1	WP_011134276.1	2	89
**129**	**β-PB**	***Hydrogenophaga taeniospiralis***	**ht**	**2K1**	**NZ_BCWR01000011.1**	**htA**	**WP_068173291.1**	1	**89**
**130**	**β-PB**	***Hydrogenophaga taeniospiralis***	**ht**	**2K1**	**NZ_BCWR01000011.1**	**htB**	**WP_068173293.1**	1	**90**
**131**	**β-PB**	***Hydrogenophaga taeniospiralis***	**ht**	**2K1**	**NZ_BCWR01000011.1**	**htC**	**WP_068173295.1**	1	**108**
**132**	**β-PB**	***Hydrogenophaga taeniospiralis***	**ht**	**2K1**	**NZ_BCWR01000011.1**	**htD**	**WP_068173297.1**	1	**79**
133	β-PB	*Ralstonia syzygii*	ras	R24	NZ_CP115945	rasA	WP_197334379.1	1	106
134	β-PB	*Ralstonia syzygii*	ras	R24	NZ_CP115945	rasB	WP_197334380.1	1	107
135	β-PB	*Ralstonia syzygii*	ras	R24	NZ_CP115945	rasC	WP_197334381.1	1	107
136	β-PB	*Ralstonia syzygii*	ras	R24	NZ_CP115945	rasD	WP_197334382.1	1	108
137	γ-PB	*Aliikangiella marina*	alm	GYP-15	NZ_VIKR01000001	almA	WP_210247075.1	ungrouped	88
138	γ-PB	*Aliikangiella marina*	alm	GYP-15	NZ_VIKR01000001	almB	WP_142887794.1	ungrouped	72
139	γ-PB	*Aliikangiella marina*	alm	GYP-15	NZ_VIKR01000001	almC	WP_142887795.1	ungrouped	94
140	γ-PB	*Aliikangiella marina*	alm	GYP-15	NZ_VIKR01000001	almD	WP_142887796.1	ungrouped	88
141	γ-PB	*Aliikangiella marina*	alm	GYP-15	NZ_VIKR01000001	almE	WP_210247076.1	ungrouped	89
**142**	**γ-PB**	***Caedibacter taeniospiralis***	**ct**	**51K**	**NZ_PGGB02000018.1**	**ctA**	**WP_011178428.1**	ungrouped	**93**
**143**	**γ-PB**	***Caedibacter taeniospiralis***	**ct**	**51K**	**NZ_PGGB02000018.1**	**ctB**	**WP_011178427.1**	ungrouped	**98**
**144**	**γ-PB**	***Caedibacter taeniospiralis***	**ct**	**51K**	**NZ_PGGB02000018.1**	**ctD**	**WP_011178426.1**	3	**92**
145	γ-PB	*Exilibacterium tricleocarpae*	ext	R142	NZ_ML660094	extB	WP_142905012.1	ungrouped	88
146	γ-PB	*Exilibacterium tricleocarpae*	ext	R142	NZ_ML660094	extC	WP_142905009.1	ungrouped	88
147	γ-PB	*Exilibacterium tricleocarpae*	ext	R142	NZ_ML660094	extD	WP_142905008.1	ungrouped	114
148	γ-PB	*Exilibacterium tricleocarpae*	ext	R142	NZ_ML660094	extE	WP_142905007.1	3	105
149	γ-PB	*Lysobacter enzymogenes*	lye	M497-1	NZ_AP014940	lyeA	WP_074861646.1	3	81
150	γ-PB	*Lysobacter enzymogenes*	lye	M497-1	NZ_AP014940	lyeC	WP_074861650.1	2	80
151	γ-PB	*Lysobacter enzymogenes*	lye	M497-1	NZ_AP014940	lyeD	WP_096376583.1	2	74
**152**	**γ-PB**	***Marinomonas mediterranea***	**mm**	**MMB-1**	**NC_015276.1**	**mmA**	**WP_013661859.1**	1	**72**
**153**	**γ-PB**	***Marinomonas mediterranea***	**mm**	**MMB-1**	**NC_015276.1**	**mmB**	**WP_013661858.1**	1	**68**
**154**	**γ-PB**	***Marinomonas mediterranea***	**mm**	**MMB-1**	**NC_015276.1**	**mmC**	**WP_013661857.1**	1	**77**
**155**	**γ-PB**	***Marinomonas mediterranea***	**mm**	**MMB-1**	**NC_015276.1**	**mmD**	**WP_013661856.1**	1	**106**
**156**	**γ-PB**	***Marinomonas mediterranea***	**mm**	**MMB-1**	**NC_015276.1**	**mmA1**	**WP_013661812.1**	ungrouped	**112**
**157**	**γ-PB**	***Marinomonas mediterranea***	**mm**	**MMB-1**	**NC_015276.1**	**mmB1**	**WP_013661811.1**	1	**96**
**158**	**γ-PB**	***Marinomonas mediterranea***	**mm**	**MMB-1**	**NC_015276.1**	**mmC1**	**WP_013661810.1**	1	**89**
**159**	**γ-PB**	***Marinomonas mediterranea***	**mm**	**MMB-1**	**NC_015276.1**	**mmD1**	**WP_013661809.1**	1	**88**
**160**	**γ-PB**	***Marinomonas mediterranea***	**mm**	**MMB-1**	**NC_015276.1**	**mmE1**	**WP_013661808.1**	1	**90**
**161**	**γ-PB**	***Marinomonas mediterranea***	**mm**	**MMB-1**	**NC_015276.1**	**mmH1**	**WP_013661804.1**	1	**97**
162	γ-PB	*Photobacterium lipolyticum*	phl	DSM 16190	NZ_PYMC01000001	phlA	WP_107281313.1	1	89
163	γ-PB	*Photobacterium lipolyticum*	phl	DSM 16190	NZ_PYMC01000001	phlB	WP_107281312.1	1	88
164	γ-PB	*Photobacterium lipolyticum*	phl	DSM 16190	NZ_PYMC01000001	phlC	WP_107281311.1	1	92
165	γ-PB	*Photobacterium lipolyticum*	phl	DSM 16190	NZ_PYMC01000001	phlD	WP_107281310.1	1	120
166	γ-PB	*Photobacterium lipolyticum*	phl	DSM 16190	NZ_PYMC01000001	phlE	WP_107281309.1	1	105
167	γ-PB	*Photobacterium lipolyticum*	phl	DSM 16190	NZ_PYMC01000001	phlF	WP_107281308.1	1	103
168	γ-PB	*Pseudoalteromonas luteoviolacea*	psl	H2	NZ_CM017105	pslA	WP_142046526.1	1	118
169	γ-PB	*Pseudoalteromonas luteoviolacea*	psl	H2	NZ_VIGJ01000001	pslA1	WP_063358517.1	1	165
170	γ-PB	*Pseudoalteromonas luteoviolacea*	psl	H2	NZ_VIGJ01000001	pslB1	WP_063358518.1	1	99
171	γ-PB	*Pseudoalteromonas luteoviolacea*	psl	H2	NZ_VIGJ01000001	pslC1	WP_023401227.1	1	89
172	γ-PB	*Pseudoalteromonas luteoviolacea*	psl	H2	NZ_VIGJ01000001	pslD1	WP_063358519.1	1	88
173	γ-PB	*Pseudoalteromonas luteoviolacea*	psl	H2	NZ_VIGJ01000001	pslH1	WP_063358523.1	1	88
174	γ-PB	*Pseudomonas aeruginosa*	pa	PA14	NZ_CP127126.1	paA	WP_003090893.1	3	66
175	γ-PB	*Pseudomonas aeruginosa*	pa	PA14	NZ_CP127126.1	paC	WP_003108980.1	2	87
176	γ-PB	*Pseudomonas aeruginosa*	pa	PA14	NZ_CP127126.1	paD	WP_003108983.1	2	86
177	γ-PB	*Pseudomonas antarctica*	psa	LMG 22709	NZ_LT629704.1	psaA	WP_003187543.1	3	84
178	γ-PB	*Pseudomonas antarctica*	psa	LMG 22709	NZ_LT629704.1	psaC	WP_083355834.1	2	87
179	γ-PB	*Pseudomonas antarctica*	psa	LMG 22709	NZ_LT629704.1	psaD	WP_078731054.1	2	92

**180**	**γ-PB**	***Pseudomonas chlororaphis***	**pc**	**DSM 50083**	**CP027712.1**	**pcA**	**AZC99429.1**	3	**80**
**181**	**γ-PB**	***Pseudomonas chlororaphis***	**pc**	**DSM 50083**	**CP027712.1**	**pcC**	**AZC99432.1**	2	**106**
**182**	**γ-PB**	***Pseudomonas chlororaphis***	**pc**	**DSM 50083**	**CP027712.1**	**pcD**	**AZC99433.1**	2	**106**
183	γ-PB	*Pseudomonas fluorescens*	pf	A506	CP003041	pfA	AFJ56504.1	3	75
184	γ-PB	*Pseudomonas fluorescens*	pf	A506	CP003041	pfC	AFJ59387.1	2	104
185	γ-PB	*Pseudomonas fluorescens*	pf	A506	CP003041	pfD	AFJ56830.1	2	104
186	γ-PB	*Pseudomonas libanensis*	pl	DSM 17149	NZ_JYLH01000014	plA	WP_003187543.1	3	75
187	γ-PB	*Pseudomonas libanensis*	pl	DSM 17149	NZ_JYLH01000014	plD	WP_057010494.1	2	104
188	γ-PB	*Pseudomonas libanensis*	pl	DSM 17149	NZ_JYLH01000014	plE	WP_057010495.1	2	103
189	γ-PB	*Pseudomonas mucidolens*	psm	LMG 2223	NZ_LT629802.1	psmA	WP_084381397.1	3	75
190	γ-PB	*Pseudomonas mucidolens*	psm	LMG 2223	NZ_LT629802.1	psmC	WP_084381393.1	2	104
191	γ-PB	*Pseudomonas mucidolens*	psm	LMG 2223	NZ_LT629802.1	psmD	WP_084381392.1	2	104
192	γ-PB	*Pseudomonas palleroniana*	psp	LMG 23076	GCF_900105975.1	pspA	WP_003187543.1	3	75
193	γ-PB	*Pseudomonas palleroniana*	psp	LMG 23076	GCF_900105975.1	pspC	WP_060755527.1	2	104
194	γ-PB	*Pseudomonas palleroniana*	psp	LMG 23076	GCF_900105975.1	pspD	WP_090367596.1	2	104
195	γ-PB	*Pseudomonas protegens*	pp	Pf-5	NC_004129.6	ppA	WP_011058560.1	3	75
196	γ-PB	*Pseudomonas protegens*	pp	Pf-5	NC_004129.6	ppC	WP_011058563.1	2	104
197	γ-PB	*Pseudomonas protegens*	pp	Pf-5	NC_004129.6	ppD	WP_011058564.1	2	104
198	γ-PB	*Pseudomonas synxantha*	ps	BG33R	NZ_CM001514	psA	WP_003187543.1	3	75
199	γ-PB	*Pseudomonas synxantha*	ps	BG33R	NZ_CM001514	psD	WP_005783676.1	2	104
200	γ-PB	*Pseudomonas synxantha*	ps	BG33R	NZ_CM001514	psE	WP_003187551.1	2	104
201	γ-PB	*Shewanella denitrificans*	shd	OS217	NC_007954.1	shdA	WP_011497572.1	1	75
202	γ-PB	*Shewanella denitrificans*	shd	OS217	NC_007954.1	shdB	WP_011497571.1	1	104
203	γ-PB	*Shewanella denitrificans*	shd	OS217	NC_007954.1	shdC	WP_011497570.1	1	104
204	γ-PB	*Shewanella denitrificans*	shd	OS217	NC_007954.1	shdD	WP_011497569.1	1	75
205	γ-PB	*Stenotrophomonas maltophilia*	stm	R551-3	NC_011071	stmA	WP_006368766.1	1	104
206	γ-PB	*Stenotrophomonas maltophilia*	stm	R551-3	NC_011071	stmB	WP_006368769.1	1	104
207	γ-PB	*Stenotrophomonas maltophilia*	stm	R551-3	NC_011071	stmC	WP_006368771.1	1	77
208	γ-PB	*Stenotrophomonas maltophilia*	stm	R551-3	NC_011071	stmD	WP_005416651.1	1	88
209	γ-PB	*Vibrio coralliilyticus*	vic	ATCC BAA-450	NZ_ACZN01000016	vicA	WP_006960107.1	1	88
210	γ-PB	*Vibrio coralliilyticus*	vic	ATCC BAA-450	NZ_ACZN01000016	vicB	WP_006960108.1	1	87
211	γ-PB	*Vibrio coralliilyticus*	vic	ATCC BAA-450	NZ_ACZN01000016	vicC	WP_006960109.1	1	91
212	γ-PB	*Vibrio coralliilyticus*	vic	ATCC BAA-450	NZ_ACZN01000016	vicH	WP_006960114.1	1	110
213	γ-PB	*Vibrio coralliilyticus*	vic	ATCC BAA-450	NZ_ACZN01000016	vicI	WP_006960115.1	3	66
214	γ-PB	*Vibrio nigripulchritudo*	vin	SFn1	NC_022528.1	vinA	WP_004398404.1	1	88
215	γ-PB	*Vibrio nigripulchritudo*	vin	SFn1	NC_022528.1	vinB	WP_004398403.1	1	86
216	γ-PB	*Vibrio nigripulchritudo*	vin	SFn1	NC_022528.1	vinC	WP_004398402.1	1	90
217	γ-PB	*Vibrio nigripulchritudo*	vin	SFn1	NC_022528.1	vinD	WP_022551678.1	1	63
218	γ-PB	*Vibrio nigripulchritudo*	vin	SFn1	NC_022528.1	vinG	WP_022551675.1	1	66
219	γ-PB	*Vibrio nigripulchritudo*	vin	SFn1	NC_022528.1	vinH	WP_004398396.1	3	89
220	γ-PB	*Xanthomonas citri*	xac	pv. citri str. 306	NC_003919	xacA	WP_011051975.1	1	88
221	γ-PB	*Xanthomonas citri*	xac	pv. citri str. 306	NC_003919	xacB	WP_011051976.1	1	89
222	γ-PB	*Xanthomonas citri*	xac	pv. citri str. 306	NC_003919	xacC	WP_011051977.1	1	90
223	γ-PB	*Xanthomonas citri*	xac	pv. citri str. 306	NC_003919	xacD	WP_011051978.1	1	62
224	δ-PB	*Corallococcus exiguus*	coe	NCCRE002	NZ_JAFIMT010000001	coeA	WP_172816348.1	1	89
225	δ-PB	*Corallococcus exiguus*	coe	NCCRE002	NZ_JAFIMT010000001	coeB	WP_206877566.1	1	89
226	δ-PB	*Corallococcus exiguus*	coe	NCCRE002	NZ_JAFIMT010000001	coeC	WP_172816347.1	3	89
227	δ-PB	*Corallococcus exiguus*	coe	NCCRE002	NZ_JAFIMT010000001	coeD	WP_172816344.1	1	94
228	δ-PB	*Corallococcus exiguus*	coe	NCCRE002	NZ_JAFIMT010000001	coeE	WP_206877571.1	1	90
229	δ-PB	*Corallococcus exiguus*	coe	NCCRE002	NZ_JAFIMT010000001	coeF	WP_172816342.1	1	89
230	δ-PB	*Corallococcus exiguus*	coe	NCCRE002	NZ_JAFIMT010000001	coeG	WP_172816341.1	3	100
231	δ-PB	*Desulfatibacillum aliphaticivorans*	dea	AK-01	NC_011768	deaA	WP_015949401.1	1	88
232	δ-PB	*Desulfatibacillum aliphaticivorans*	dea	AK-01	NC_011768	deaB	WP_015949400.1	1	93
233	δ-PB	*Desulfatibacillum aliphaticivorans*	dea	AK-01	NC_011768	deaC	WP_015949399.1	1	84
234	δ-PB	*Desulfatibacillum aliphaticivorans*	dea	AK-01	NC_011768	deaD	WP_015949398.1	1	88
235	δ-PB	*Desulfatibacillum aliphaticivorans*	dea	AK-01	NC_011768	deaE	WP_015949397.1	1	93
236	δ-PB	*Desulfatibacillum aliphaticivorans*	dea	AK-01	NC_011768	deaF	WP_015949396.1	1	99
237	δ-PB	*Desulfatibacillum aliphaticivorans*	dea	AK-01	NC_011768	deaG	WP_015949395.1	1	76
238	δ-PB	*Plesiocystis pacifica*	plp	SIR-1	NZ_ABCS01000129	plpB	WP_006976347.1	ungrouped	74
239	δ-PB	*Plesiocystis pacifica*	plp	SIR-1	NZ_ABCS01000129	plpC	WP_006976346.1	ungrouped	102

240	δ-PB	*Plesiocystis pacifica*	plp	SIR-1	NZ_ABCS01000129	plpD	WP_006976345.1	ungrouped	103
241	δ-PB	*Plesiocystis pacifica*	plp	SIR-1	NZ_ABCS01000129	plpE	WP_006976344.1	ungrouped	106
242	δ-PB	*Plesiocystis pacifica*	plp	SIR-1	NZ_ABCS01000129	plpF	WP_006976343.1	ungrouped	106
243	δ-PB	*Plesiocystis pacifica*	plp	SIR-1	NZ_ABCS01000129	plpG	WP_006976342.1	ungrouped	102
244	δ-PB	*Plesiocystis pacifica*	plp	SIR-1	NZ_ABCS01000129	plpH	WP_006976341.1	ungrouped	111
245	δ-PB	*Plesiocystis pacifica*	plp	SIR-1	NZ_ABCS01000129	plpJ	WP_006976334.1	3	61
246	CB	*Anabaena azotica*	ana	FACHB-119	NZ_JACJSG010000020	anaA	WP_190474086.1	ungrouped	112
247	CB	*Anabaena azotica*	ana	FACHB-119	NZ_JACJSG010000020	anaB	WP_190474083.1	ungrouped	115
248	CB	*Anabaena azotica*	ana	FACHB-119	NZ_JACJSG010000020	anaC	WP_190474081.1	ungrouped	109
249	CB	*Anabaena azotica*	ana	FACHB-119	NZ_JACJSG010000020	anaD	WP_190474080.1	ungrouped	106
250	CB	*Anabaena azotica*	ana	FACHB-119	NZ_JACJSG010000020	anaE	WP_190474078.1	ungrouped	109
251	CB	*Aphanothece sacrum*	aps	FPU1	NZ_BDQK01000015	apsA	WP_124974426.1	1B	146
252	CB	*Aphanothece sacrum*	aps	FPU1	NZ_BDQK01000015	apsB	WP_124974418.1	1B	76
253	CB	*Aphanothece sacrum*	aps	FPU1	NZ_BDQK01000015	apsC	WP_124974420.1	1B	271
254	CB	*Aphanothece sacrum*	aps	FPU1	NZ_BDQK01000015	apsD	WP_124974422.1	1B	97
255	CB	*Aphanothece sacrum*	aps	FPU1	NZ_BDQK01000015	apsE	WP_124974424.1	1B	75
256	CB	*Aphanothece sacrum*	aps	FPU1	NZ_BDQK01000015	apsF	WP_227873497.1	ungrouped	118
257	CB	*Aphanothece sacrum*	aps	FPU1	NZ_BDQK01000015	apsG	WP_125061154.1	3	133
258	CB	*Aphanothece sacrum*	aps	FPU1	NZ_BDQK01000015	apsH	WP_124974416.1	3	101
259	CB	*Aphanothece sacrum*	aps	FPU1	NZ_BDQK01000015	apsJ	WP_124974412.1	ungrouped	105
260	CB	*Aphanothece sacrum*	aps	FPU1	NZ_BDQK01000015	apsK	WP_124974410.1	3	106
261	FB	*Chryseobacterium indologenes*	chi	PgBE177	NZ_CP074372	chiA	WP_034697211.1	3	72
262	FB	*Chryseobacterium indologenes*	chi	PgBE177	NZ_CP074372	chiB	WP_213280166.1	ungrouped	88
263	FB	*Chryseobacterium indologenes*	chi	PgBE177	NZ_CP074372	chiC	WP_047378383.1	ungrouped	87
264	FB	*Chryseobacterium indologenes*	chi	PgBE177	NZ_CP074372	chiD	WP_249413657.1	ungrouped	86
265	FB	*Chryseobacterium indologenes*	chi	PgBE177	NZ_CP074372	chiE	WP_223599388.1	ungrouped	71
266	FB	*Chryseobacterium indologenes*	chi	PgBE177	NZ_CP074372	chiF	WP_047421299.1	ungrouped	75
267	FB	*Chryseobacterium indologenes*	chi	PgBE177	NZ_CP074372	chiG	WP_047378387.1	ungrouped	85
268	FB	*Kordia algicida*	koa	OT-1	NZ_DS544873	koaA	WP_007093070.1	3	85
269	FB	*Kordia algicida*	koa	OT-1	NZ_DS544873	koaC	WP_007093065.1	ungrouped	85
270	FB	*Kordia algicida*	koa	OT-1	NZ_DS544873	koaD	WP_007093064.1	ungrouped	85
271	FB	*Kordia algicida*	koa	OT-1	NZ_DS544873	koaE	WP_007093063.1	ungrouped	87
