# Supplementary material for: Bacterial R-bodies with common morphologies and unrolling dynamics are phylogenetically scattered, indicating extensive lateral gene transfer and wide application potential
Source: Appl Environ Microbiol. 2026 Sep 11;92(10):e00864-26. doi: 10.1128/aem.00864-26 (PMC13689365; doi:10.1128/aem.00864-26)
Supplement: Supplementary file 2 [file aem.00864-26-s0001.docx]

**FIG S1**: Rectangular phylogenetic maximum likelihood tree based on sequences of 62 different bacterial strains with homology to RebB from *Cb. taeniospiralis*. Reb proteins of the species studied here are highlighted. For labelling of proteins, 'Reb' was omitted for readability (e.g. cvA is cvRebA). Numbers on branches show ultra-fast bootstrap values. Scale bar: the estimated proportional sequence divergence. Abbreviations: ct, *Cb. taeniospiralis*; cv, *Cm. varicaedens*; ra, *Ro. alexandrii*; pc, *Ps. chlororaphis*; aa, *Ac. avenae*; ht, *Hy. taeniospiralis*; mm, *Ma. mediterranea*.

**FIG S2** Transmission electron microscopy images. (A) Tubular phage-like structures in the *Ps. chlororaphis* isolate. (B) *Ps. chlororaphis* R-body with extreme height. (C) Transition between two distinct layers (arrow) seen on an R-body isolated from *Ps. chlororaphis*. (D) to (F) Ultra-thin sections of high-pressure frozen *Cm. varicaedens* cells with R-bodies in the macronucleus of *P. caudatum*. The white arrows mark the sheath. The orange arrow marks where either the sheath or the outer R-body lamella appears connected to the cell membrane.

**TABLE S1** Complete list of protein sequences used for the phylogenetic outline. Sequences of the seven species used in this paper are highlighted in bold. Abbr. = abbreviation, PB = proteobacteria, CB = cyanobacteria, FB = flavobacteria.

**VID S1** Light microscopic video recordings of R-bodies isolated from *Cm. varicaedens* (A-C), *Ro. alexandrii* (D), *Ps. chlororaphis* (E) and *Ac. avenae* (F) unrolling acid-induced (A, D-F) or alkali-induced (B-C). Videos were recorded in Full HD with 120 frames per second and slowed down 4x (A-B, E-F) or recorded in 4K Crop with 60 frames per second without slow-down (C-D). Videos were scaled to 1,080 px.

**Supplementary Materials & Methods**

**Procedures used for the isolation of R-bodies**

***(A) Large-scale isolation R-bodies of Cm. varicaedens.*** 20-40 L paramecial culture were starved for 9-11 days before isolation. The culture was filtered through 8 layers of gauze, then concentrated using a dairy centrifuge. The pellet was washed and then passed once more through 8 gauze layers. The filtrate was centrifuged for 3 min at 300 × g, after which the supernatant was removed using a vacuum pump. The pellets were homogenized using a glass homogenizer in Buffer A (50 mM TrisHCl pH 8.5), then subjected to four freeze-thaw cycles, followed by incubation in Buffer A containing 2 % (w/v) octyl-polyoxyethylene (OPOE, Bachem, CH,) at 4°C for 48 h. Finally, the detergent-extracted R-bodies were washed in Buffer A and then suspended in a small volume (usually about 2 ml) of the same buffer. The resuspended R-body preparation was then stored at 4°C or flash-frozen in liquid nitrogen prior to storage at -80°C.

(B) ***Isolation of R-body proteins from medium-scale (400 ml) cultures****.* Used for *Ac. avenae*, *Ma. mediterranea*, and *Hy. taeniospiralis*. The bacteria were grown in their respective media as listed above, and if appropriate (*Ac. avenae*, *Ma. mediterranea*) starved for 6-8 days before isolation. After harvesting by centrifugation, the pellets were suspended in 7.5 ml Buffer A and then subjected to six freeze-thaw cycles for initial cell breakage. Hen egg white lysozyme (HEWL, Sigma-Aldrich GmbH) and EDTA were added to a final concentration of 0.13 mg/ml and 33 mM, respectively, and then the complete cocktail was incubated first at 37°C for 1 h, and then overnight at room temperature. The lysed cells were then subjected to further freeze-thaw cycles (5x) and the chromosomal DNA released after breakage was digested by the addition of DNAase I (9 μg/ml) and 8 mM MgCl_2_ by an initial incubation for 30 min at 37°C and subsequently an overnight incubation at room temperature. The breakage lysates were further solubilized for 3 days at room temperature using a detergent mixture containing 1% (v/v) Triton X-100 and 1% (w/v) SDS. After centrifugation, the pellets were washed twice by centrifugation using Buffer A and then purified through an additional centrifugation step at 64 × g (*Ac. avenae*) or 240 × g (*Ma. mediterranea*). The supernatant (which contains the R-bodies) was then centrifuged, and the final pellet was suspended in a small volume (usually 1 ml) of Buffer A prior to storage as in (A).

(C) ***Isolation of R-bodies from small-scale cultures (40 ml).*** Used for cultures of *Hy. taeniospiralis* and *Ma. mediterranea*, respectively). The procedure was similar to (B), except that Buffer A was replaced with 50 mM TrisHCl pH 8.5 containing 100 mM NaCl and 1 mM EDTA (Buffer B), only four freeze-thaw cycles were performed, and the amounts of HEWL and DNAase I were modified (0.3 mg/ml and 8 μg/ml, respectively). Also, the long incubation steps at room temperature were omitted. An important difference to the procedure, however, is that the detergent solubilization step was SDS-free, employing only Triton X-100 (1.2% (v/v) and 0.9% (v/v) for *Hy. taeniospiralis* and *Ma. mediterranea*, respectively) with an overnight incubation at 4°C. R-bodies were then washed and harvested by centrifugation as in (B).

(D) ***Isolation of R-bodies from agar plate cultures.*** Used for *Ro. alexandrii*, *Ac. avenae*, *Ps. chlororaphis*. In general, for each species, two agar plate cultures of each species were scraped up and suspended in either Buffer B (*Ro. alexandrii* and *Ac. avenae*) or 1 ml medium *(Ps. chlororaphis)*. For *Ro. alexandrii* and *Ac. avenae,* the plates were illuminated with UV-A light (5 W, 365 nm at about 8 cm for 90 s) and then incubated at 25 - 26°C overnight before harvesting. The pooled suspensions were subjected to freeze-thaw cycles (4x) and then incubated with 1.6 mg/ml HEWL for 60 min. at 37°C. The pools from *Ro. alexandrii* (0.65% (v/v) Triton X-100) and *Ps. chlororaphis* (0.5% (v/v) Triton X-100) were subjected to an initial detergent solubilization step (indicated in parentheses) for 1 h at 37°C. The partially solubilized lysates of *Ro. alexandrii* and *Ps. chlororaphis* were fragmented further by ultrasonication, using an ultrasonication tip (Bandelin Sonopuls Homogenisator UW3100, Germany), for 10 min. using a 5 s duty cycle or an ultrasonic bath for 2 h using a 10 min duty cycle respectively. After subsequent incubation with DNAase I (8 μg/ml) in Buffer B containing 7.5 mM MgCl_2_ (10 mM CaCl_2_ was also added to the lysate from *Ac. avenae*), the lysates were pelleted by centrifugation, resuspended in Buffer A, and then subjected to a second detergent solubilization step using either 0.65% (v/v) (*Ro. alexandrii*), 1.2% (v/v) (*Ac. avenae*) or 0.5% (v/v) (*Ps. chlororaphis*) Triton X-100. Finally, the pellets were washed twice by centrifugation, using Buffer A for each resuspension step. Isolated R-bodies of *Ro. alexandrii* were purified with a further centrifugation step at 68 × g as in (B). The final pellet was resuspended in about 1 ml Buffer A and stored as described in (A).

**Preparation for electron microscopy**

Isolated R-bodies were prepared for electron microscopy (EM) using negative contrast imaging as follows. 5 µl of the R-body isolate was adsorbed for 5 min onto a copper mesh grid coated with a thin Pioloform film. Subsequently, the excess liquid was removed with a filter paper edge and the grid was placed upside down onto a drop of 1 % (w/v) phosphotungstic acid. After about for 5 s the acid was removed from one side with a filter paper edge. If required, R-bodies in the isolate were unrolled by diluting them 1:1 in 1 M HCl before adsorbing them to the grid.

For the preparation of whole bacteria or paramecial cells for EM, R-body production was induced, and non-endosymbiotic cells were harvested as described above. Paramecia were concentrated by centrifugation with an oil centrifuge as described above and suspended in ~10 % (w/v) dextran solution. Cells were vitrified using a high-pressure freezer (Leica ICE) employing either two gold carriers for paramecia or aluminum carriers for bacteria. Frozen samples (1.0 - 1.5 ml each) were then freeze-substituted using a Leica/Reichert AFS apparatus at a starting temperature of -90°C. The cells were covered with about 150 µl of an acetone staining solution containing 2.5 % (v/v) glutaraldehyde, 1 % (w/v) osmium tetroxide, 0.1 % (w/v) uranyl acetate and ~10 % (v/v) water and 86.4% acetone. For *Ro. alexandrii*, *Ps. chlororaphis* and *Ac. avenae*, no glutaraldehyde was used. The freeze-substitution for paramecia kept the temperature at -90°C for 18 min before heating (heating rate 30°C/h) until 0°C was reached. For non-endosymbiotic bacteria, the temperature was maintained at -90°C for 72 h before heating to -30°C (heating rate 1°C/h). After 8 h at
-30°C, the solution was washed with water-free acetone before heating to 0°C within 24 h (heating rate 1.25°C/h).

After freeze substitution, the paramecial samples were transferred to room temperature, and then then washed four times with acetone (each wash with a 10 min incubation). Carriers were removed. Embedding was performed by the addition of Spurr resin at 1:2 volume ratio to the acetone suspension. The suspension was mixed, capped, and then incubated for 15 min at room temperature. Finally, the tube was opened and the acetone allowed to evaporate overnight. The remaining resin was replenished twice, each time with a 1 h incubation, and then hardened at 60°C. Non-endosymbiotic bacteria were also processed as described, but only washed twice with acetone, and incubated longer (more than 1 h) after adding the resin.

The hardened resin blocks were first trimmed by hand and then ultra-thin sectioned into 60 - 70 nm thick slices with an ultra-microtome (Leica Ultracut UCT) using either a Diatome or Drukker 45° diamond knife. The thin slices were collected in the water (Milli-Q)-filled knife trough of the knife. Slices were treated with vapor from a 50 % (v/v) xylene/chloroform reservoir and then transferred onto Pioloform film-coated copper mesh grids.
